# Mechanical signal modulates prostate cancer immune escape by USP8-mediated ubiquitination-dependent degradation of PD-L1 and MHC-1

**DOI:** 10.1038/s41419-025-07736-4

**Published:** 2025-05-23

**Authors:** Zhi-Bin Ke, Jia-Yin Chen, Yu-Ting Xue, Bin Lin, Qi Huang, Xu-Yun Huang, Dong-Ning Chen, Shao-Hao Chen, Xiao-Jian Ye, Qing-Shui Zheng, Yong Wei, Xue-Yi Xue, Ning Xu

**Affiliations:** 1 Department of Urology, Urology Research Institute, the First Affiliated Hospital, Fujian Medical University, Fuzhou, 350005 China; 2 Department of Urology, National Regional Medical Center, Binhai Campus of the First Affiliated Hospital, Fujian Medical University, Fuzhou, 350212 China; 3 Department of Ultrasonography, the First Affiliated Hospital, Fujian Medical University, Fuzhou, 350005 China; 4 Fujian Key Laboratory of Precision Medicine for Cancer, the First Affiliated Hospital, Fujian Medical University, Fuzhou, 350005 China

**Keywords:** Urological cancer, Ubiquitylation, Cancer microenvironment

## Abstract

The tumor environment of prostate cancer (PCa) tissues of high Gleason score has been proved to be more immune suppressive and has higher extracellular matrix (ECM) stiffness, but whether ECM mechanical stiffness is the cause of higher ability of invasiveness and immune escape of PCa with high Gleason score remains uncertain. In this study, we showed that higher polyacrylamide hydrogels (PAAG) stiffness resulted in the progression and immune escape of PCa via integrin β1/FAK/YAP axis. The translocation of YAP into cell nucleus to bind to TEAD2 promoted the transcriptional activation of USP8. NBR1 could be ubiquitinated, and then degraded, via interacting with P62/SQSTM1 and through autophagy-lysosome pathway. Increased expression of USP8 promoted the abundance of NBR1 via K63-linked de-ubiquitination and PD-L1 via K48-linked de-ubiquitination in response to high PAAG stiffness. NBR1-mediated selective autophagy accelerated the degradation of MHC-1 of PCa. The USP8 inhibitor presented a potential application value in sensitizing immunotherapy of PCa. Taken together, we identified a USP8-mediated de-ubiquitination mechanism that involves in the process of high PAAG stiffness-mediated high expression of PD-L1 and low expression of MHC-1 of PCa cells, which provided a rationale of immunotherapy sensitization of PCa via USP8 inhibition.

## Introduction

Prostate cancer (PCa) is regarded as the most frequent cancer in men around the world [1]. Despite initial response to therapy, unfortunately, some PCa patients eventually develop more aggressive forms resistant to hormonotherapy, radiotherapy and chemotherapy, leading to poor prognosis [2]. Recently, immunotherapy has been proved as an effective therapeutic option for improving the survival of cancer patients [3]. However, PCa usually has an immunosuppressive tumor microenvironment and is considered as a “cold tumor”, resulting in the inefficacy of immunotherapy, especially those with higher Gleason score [4, 5]. The specific mechanism of immune evasion and resistance to immunotherapy remains unclear in PCa.

In the process of immune surveillance, major histocompatibility complex class I (MHC-I)/T cell receptor (TCR) complex is an important molecule that plays a key role in antigen presentation and immune signal transmission, and is an important target for cytotoxic T lymphocytes (CTLs) to recognize and kill tumor cells [6]. Immune checkpoint (for example, PD-L1, PD1, and CTLA-4) is a class of immune suppression molecules expressed on tumor or immune cells and can regulate the degree of immune activation [7]. In tumor tissues, abnormal signals of immune checkpoint or MHC-I/TCR complex would weaken the immune response of immune system and promote the immune evasion of cancer cells [3, 7]. The expression level of PD-L1 was proved to be positively correlated with Gleason score of PCa [8], and MHC-1 was down-regulated in tissues of high Gleason score than those of low Gleason score [9], which is one of the causes that high Gleason score tissues have more suppressive immune environment. However, the specific molecular mechanism of differential expression of PD-L1 and MHC-1 in PCa tissues with various Gleason score was less studied.

The extracellular matrix (ECM) is a non-cellular scaffold in tumor microenvironment of tumors, which could provide nests for tumor and stroma cells [10]. The cancer ECM is mainly established by collagens, elastin, proteoglycans, glycoproteins, laminins, fibronectins and other matrix proteins. The unbalance between ECM deposition and degradation results in the promotion of malignant phenotypes of cancer [10]. High level of ECM stiffness has been reported to enhance the proliferation, migration, and invasion of cancer cells. It has also been reported that higher Gleason score represents higher ECM mechanical stiffness, and that PCa with higher Gleason score is more susceptible to immune escape [11]. However, whether ECM mechanical stiffness is the cause of higher ability of invasiveness and immune escape of PCa with high Gleason score remains uncertain.

In this study, we aimed to investigate the impact of ECM-derived mechanical stiffness on PCa progression and immune evasion and the potential molecular mechanisms. We elucidated that ECM-derived biomechanical stiffness could inhibit Hippo signaling to induce the progression and immune evasion of PCa via integrin β1/FAK/YAP axis. Furthermore, we revealed that USP8 was a major downstream target protein of YAP/TEAD2-mediated transcriptional regulation and USP8-mediated de-ubiquitination is essentially responsible for the MHC-1 degradation and PD-L1 abundance of PCa, which is the leading molecular mechanism of ECM-derived PCa progression and immune evasion. Our results highlight the role of biomechanical signaling in ECM-induced tumor progression and immune evasion of PCa and the underlying therapeutic value of USP8 inhibition in immunotherapy sensitization.

## Material and methods

### Cell culture

PC-3, DU145, RM-1, and HEK-293T cells were purchased from Procell Life Science & Technology Co., Ltd (Wuhan, China). PC-3 cells were cultured in F-12K medium supplemented with 10% fetal bovine serum. DU145 cells were cultured in MEM medium with 10% fetal bovine serum. RM-1 and HEK-293T cells were cultured in DMEM medium supplemented with 10% fetal bovine serum. All cells were cultured at 37 °C with 5% CO_2_.

### Clinical specimens

We obtained fresh PCa tissues (24 cases of Gleason score < 7, 25 cases of Gleason score = 7, and 28 cases of Gleason score > 7) for paraffin embedded wax blocks and immunohistochemical staining (IHC) from the First Affiliated Hospital of Fujian Medical University. Besides, 36 cases (12 cases of Gleason score < 7, 12 cases of Gleason score = 7, and 12 cases of Gleason score > 7) of fresh postoperative PCa tissues were obtained from the First Affiliated Hospital of Fujian Medical University for protein extraction and western blot or immunoprecipitation. Written informed consent was provided by all included patients before sample collection.

### Ultrasonic elastography for tissue stiffness assessment

Ultrasound elastography was performed to assess the stiffness of prostate cancer tissues. Patients were positioned in the left lateral decubitus position, and an ultrasound probe was inserted rectally under aseptic conditions. We used a cognitive fusion image of ultrasound, mpMRI and PSMA PET/CT to locate the region of interest (ROI) for ultrasound elastography measure before prostate biopsy. The system was switched to shear wave elastography (SWE) mode to measure tissue stiffness. The Young’s modulus (E, in kilopascals) was calculated based on shear wave velocity measurements. For each ROI, three independent measurements were obtained and averaged to ensure reproducibility. Patients were instructed to hold their breath briefly during image acquisition to maintain stability. All ultrasound examinations were performed by an experienced radiologist.

Besides, during prostate biopsy, we performed a puncture biopsy for the ROI, and obtained the Gleason score of the ROI from biopsy pathological report. After radical prostatectomy, we used postoperative large pathological section of prostate for final confirmation of the Gleason score of the ROI. If the Gleason score of the ROI was different from biopsy to radical prostatectomy, the patients was excluded from final analysis. In this study, we enrolled a total of 77 case for the measure of prostate stiffness via ultrasonic elastography (including 24 cases of Gleason < 7, 25 cases of Gleason = 7, 28 cases of Gleason > 7).

### Construction of polyacrylamide hydrogels (PAAG) model with three kinds of stiffness

Polyacrylamide hydrogels (PAAG) were made up of 40% acrylamide, 2% methylene diacrylamide, 1 M HEPES, and ddH2O (low PAAG stiffness: 150μl 40% acrylamide + 25μl 2% methylene diacrylamide + 10μl 1 M HEPES + 815μl ddH2O; medium PAAG stiffness: 368μl 40% acrylamide + 161μl 2% methylene diacrylamide + 10μl 1 M HEPES + 471μl ddH2O; high PAAG stiffness: 492μl 40% acrylamide + 401μl 2% methylene diacrylamide + 10μl 1 M HEPES + 97μl ddH2O). We did not use some proteins to functionalize the PAAG substrates. The compressive mechanical properties of PAAG were measured by texture analyzer (TAXTplus, British Stable Micro System). The elastic modulus (E) can be calculated by the formula: E = σ/ε = (F/A)/(△L/L) (F is the force applied to the PAAG to compress it; A is the actual cross-sectional area of the PAAG; ΔL is the amount of change in the height of the PAAG; L is the original height of the PAAG; F/A is the stress σ; the ratio of the shape variable to the original dimension (∆L/L) is the strain ε). The compressive elastic modulus of the sample is the slope of the initial linear region on the stress-strain curve. During the experiment, the PAAG was placed in the center of the sample table of the texture analyzer and compressed with the P36R probe at the test speed of 5 mm/min. The strain and stress changes of the sample were recorded during the test, and the stress-strain curve was drawn. The values of low, medium and high PAAG stiffness were defined as 2–3 kPa, 15–17 kPa, and 53–57 kPa, respectively.

### RNA extraction and qRT-PCR analysis

The relevant total RNA was extracted by using TRIzol reagent (Invitrogen, Carlsbad, CA). The TransScript® Green One-Step qRT-PCR SuperMix (TransGen Biotech) was used to generate cDNA. Next, we used Taq Pro Universal SYBR qPCR Master Mix (Vazyme) to detect the expression levels with the Step One PlusTM PCR System (Applied Biosystems). The expression levels were normalized based on the expression of GAPDH. The primer sequences were presented in Table S1.

### Plasmids transfection, RNA inference and lentivirus transduction

Small interfering RNA (siRNA) and overexpression plasmids were purchased from Shanghai GenePharma Co., Ltd (Shanghai, China). Lentivirus carrying shRNA targeting USP8 or NBR1 was purchased from Shanghai GENECHEM Co., Ltd (Shanghai, China). The sequences for the siRNA of YAP, TEAD1, TEAD3, TEAD4 and shRNA of USP8 and NBR1 were shown in Table S2. Cells were transfected using Lipofectamine 2000 (Invitrogen, Carlsbad, CA) in accordance with the manufacturer’s protocol. The lentivirus was used to infect PC-3 and DU145 cells accompanied with Polybrene. Cells were then cultured in medium-containing puromycin for the selection of stable clones after treated with Polybrene 48 h later.

### Immunofluorescence

Cells seeded on coverslips were fixed with 4% formaldehyde for 15 min, treated with 0.5% Triton X-100 for 5 min, blocked with 1% bovine serum albumin (BSA) for 1 h, and incubated with primary antibodies overnight at 4 °C. After 3 washes with PBS, the cells on the coverslips were incubated with fluorescent dye-conjugated anti-mouse IgG H&L or anti-rabbit IgG H&L in a dark room for 1 h, and labeled with DAPI (Beyotime, China) for 10 min. Finally, the cells were observed and images were captured under a confocal microscope.

### Immunohistochemistry

Sections of formalin-fixed, paraffin-embedded tissue (4μm thick) were deparaffinized, rehydrated, and subjected to antigen retrieval in citrate buffer (pH 6.0) at 95 °C for 20 minutes. Endogenous peroxidase activity was blocked with 3% hydrogen peroxide, followed by incubation with 5% bovine serum albumin to prevent nonspecific binding. Tissue sections were incubated overnight at 4 °C with the primary antibody, followed by incubation with an HRP-conjugated secondary antibody at room temperature for 30 minutes. Diaminobenzidine (DAB) was used as the chromogen, and hematoxylin was applied for counterstaining. The gray values to optical density (OD) values were converted by the ImageJ software (https://imagej.nih.gov/ij/, Wayne Rasband, USA). Finally, the relative expression was assessed with the average optical density (AOD). AOD=integrated optical density (IOD)/positive area.

### Hematoxylin and Eosin (HE) staining, Masson staining and Sirius Red staining

For HE staining, sections were stained with hematoxylin for 10 minutes, differentiated with 0.7% hydrochloric acid ethanol for a few seconds, and rinsed in water for 15 minutes. Eosin staining was performed for 30 seconds, followed by dehydration in 95% and 100% ethanol, clearing in xylene, and mounting with neutral balsam.

For Masson staining, sections were stained with Weigert’s iron hematoxylin for 5–10 minutes, followed by Biebrich Scarlet Acid Fuchsin for 5–10 minutes. After differentiation with 1% phosphomolybdic acid for 3–5 minutes, aniline blue or light green was applied for 5 minutes. Slides were then dehydrated in ethanol, cleared in xylene, and mounted with neutral balsam.

For Sirius Red staining, sections were incubated with 0.1% Sirius Red in saturated picric acid for 8 minutes, dehydrated through a gradient of ethanol starting from 75%, cleared in xylene, and mounted.

### Liquid chromatography-mass spectrometry analysis

4D label free quantitative proteomics and ubiquitinome were performed in stable USP8-silenced DU145 cells and control cells via Liquid chromatography-mass spectrometry analysis (LC-MS/MS). Briefly, after protein extraction and digestion, the peptides were dissolved by liquid chromatography with mobile phase A and separated by NanoElute ultra-high performance liquid phase system. The peptides are separated by an ultra-high performance liquid phase system and injected into the Capillary ion source for ionization and then analyzed by timsTOF Pro mass spectrometry. The ion source voltage was set to 1.65 kV, and the parent ion of the peptide segment and its secondary fragments were detected and analyzed using high-resolution TOF.

### Western blot analysis and Co-immunoprecipitation assay

For western blot, samples were lysed in RIPA buffer (P0013B, Beyotime), and the proteins were then quantified using a BCA assay kit (P0010, Beyotime). Proteins were separated based on their own molecular weight by 10% SDS-PAGE and transferred to PVDF membranes. The membranes were incubated at 4°C overnight with specific primary antibodies after blocking, and then incubated with HRP-conjugated goat anti rabbit or mouse IgG for 1 hour at room temperature and finally developed using ECL reagent.

For immunoprecipitation assay, samples were lysed with IP lysis buffer supplemented with a protease inhibitor cocktail and then centrifuged at 12,000 × g for 10 min. After the total protein concentrations were measured by using a BCA kit, equal amounts of lysate were incubated with IgG or specific primary antibodies plus protein A/G magnetic bead overnight at 4 °C. Then, the beads were washed three times with IP lysis buffer and prepared for western blotting.

The following is the information about antibodies used in immunoblot: anti-PD-L1 (1:1000; proteintech; 28076-1-AP), anti-USP8 (1:1000; proteintech; 67321-1-Ig), anti-NBR1 (1:1000; proteintech; 16004-1-AP), anti-β-actin (1:2000; proteintech; 20536-1-AP), anti-tubulin (1:2000; proteintech; 11224-1-AP), anti-MHC-I (1:100; Santa Cruz; sc-55582), anti-polyubiquitin (1:1000; CST; 58395), anti-K63 linkage Specific polyubiquitin (1:1000; CST; 5621S),anti-Flag (1:1000; sigma; F1804), anti-Myc (1:1000; proteintech; 16286-1-AP), anti-HA (1:1000; proteintech; 51064-2-AP), anti-His (1:1000; proteintech; 10001-0-AP), anti-integrin β1 (1:1000; immunoway; YT2367), anti-FAK (1:1000; immunoway; YT1659), anti-p-FAK (1:1000; immunoway; YP0739), anti-YAP (1:1000; immunoway; YT4924), anti-pYAP (1:1000; immunoway; YP0708), anti-PCNA (1:1000; immunoway; YT3617), anti-cyclin D1 (1:1000; immunoway; YT1173), anti-E-cadherin (1:1000; CST; 14472), anti-N-cadherin (1:1000; CST; 13116), anti-vimentin (1:1000; CST; 46173), anti-TEAD1 (1:1000; immunoway; YT4596), anti-TEAD2 (1:1000; proteintech; 21159-1-AP), anti-TEAD3 (1:1000; proteintech; 13120-1-AP), anti-TEAD4 (1:1000; proteintech; 12418-1-AP).

### Chromatin immunoprecipitation

Chromatin immunoprecipitation (ChIP) assays for DU145 and PC-3 cells were performed according to the manufacturer’s protocol of the ChIP Assay Kit (Cell Signaling Technology). Cells were treated with 1% formaldehyde for the crosslinking of protein with DNA and then sonicated to obtain DNA fragments with lengths of 500–800 base pairs. Solutions containing DNA fragments were incubated with anti-TEAD2 (proteintech; 21159-1-AP) or IgG negative control at 4 °C overnight on a rotator. Immunocomplexes were purified and used to perform reverse transcription real-time quantitative PCR for the detection of certain DNA fragments.

### In vitro and in vivo ubiquitination assay

For in vivo ubiquitination assay, cells were treated with MG132 or 3-MA for 6 h before harvested and then lysed with lysis buffer. Immunoprecipitation was carried out with relevant antibody at 4 °C overnight and the ubiquitinated proteins were purified using protein A/G magnetic beads and detected via immunoblotting using an anti-polyubiquitin antibody. For in vitro de-ubiquitination assay, Myc-NBR1 (or His-PD-L1) and HA-Ub expression vectors were co-transfected in HEK-293T cells for 24 h, and then treated with 3-MA or MG132 for 6 h, followed by immunoprecipitation to extract the ubiquitinated NBR1 or PD-L1 protein. Flag-USP8 were expressed and purified, respectively. The ubiquitinated PD-L1 or NBR1 protein was then incubated with recombinant USP8 protein in de-ubiquitination buffer (50 mmol/L Tris-HCl pH 8.0, 50 mmol/L NaCl, 1 mmol/L EDTA, 10 mmol/L DTT and 5% glycerol) for 2 h at 37 °C, respectively, and the ubiquitination level was analyzed by western blot.

### Dual-luciferase reporter assay

The luciferase reporter vectors were synthesized by Shanghai GenePharma Co., Ltd (Shanghai, China). The Luciferase assays were performed using a Dual-Lumi™ Luciferase Assay Kit (Beyotime Biotechnology, Shanghai, China) in accordance with the manufacturer’s instructions. Cells were plated in 96-well plates and co-transfected with TEAD2 plasmid or negative control together with the luciferase reporter (USP8-wt-promoter or USP8-mut-promoter) vector using Lipofectamine 2000 reagent (Invitrogen). We detected the luciferase activity 48 h later and data were normalized using Renilla luciferase activity.

### Colony formation assay, EdU staining and CCK8 assay

For colony formation assays, cells were plated into six-well plates (1 × 10^3^ cells per well) and further incubated for 2 weeks. Then, the cells were fixed with 4% formaldehyde and stained with 0.5% crystal violet. The number of colonies was then counted under a microscope.

For EdU assay, the cells were cultured in 24-well plates for 24 h. EdU assay kit (Ribobio) containing Hoechst that was used to perform the nuclei staining and incorporation assays. A fluorescent microscope was used to observe the proliferating cells.

For CCK8 assay, cells were seeded in 96 well plates (5 × 10^3^ cells per well) and cultured for 24 h, 48 h, 72 h, 96 h. The optical absorbance at 450 nm of cells was measured after adding 10 μL CCK8 reagent for 2 h.

### Transwell migration and invasion assay

24-well Transwell inserts with 8-μm pore size (Corning Inc., USA) were also applied to performed migration and invasion assay. Briefly, cells were cultured in serum-free medium overnight and approximately 3 × 10^4^ cells were resuspended in serum-free medium and added to the upper chamber. The lower chamber contained 400 μL medium containing 20% fetal bovine serum. For invasion assay, the chamber was coated with Matrigel. Cells were allowed to migrate or invade for 24 to 48 h. The cells without penetrating the membrane were removed with a cotton swab while the cells on the lower surface were fixed and stained. Five random microscopic fields were selected and photographed. When we explored the effect of different PAAG stiffness on the migration and invasion ability of PCa cells, the cells were firstly cultured in different PAAG stiffness for 48 h, and then moved to the upper chamber of 24-well Transwell inserts.

### Apoptosis assay by flow cytometry

Cell apoptosis was assessed using the Annexin V-FITC/PI Apoptosis Detection Kit (Yeasen Biotech) following the manufacturer’s protocol. Briefly, cells were harvested, washed twice with cold phosphate-buffered saline (PBS), and resuspended in 1× binding buffer at a concentration of 1–5 × 10^5^ cells/mL. Then, 5 μL of Annexin V-FITC and 10 μL of propidium iodide (PI) were added to 100 μL of the cell suspension, followed by incubation in the dark at room temperature for 15 minutes. Stained cells were analyzed using a BD Accuri C6 Plus flow cytometer (BD Biosciences). Data acquisition was performed with the system’s default settings, and results were processed using FlowJo software. Apoptotic cells were identified as Annexin V-positive/PI-negative (early apoptosis) or Annexin V-positive/PI-positive (late apoptosis).

### T cell-mediated tumor cell killing assay

CD8 + T cells were purified from peripheral blood mononuclear cells (PBMCs) of healthy volunteers. Cell purity was checked and the cells were stimulated with CD3/CD28 antibody, and co-cultured with indicated DU145 or PC-3 PCa cells, in 96-well plates at a ratio of 5:1, 10:1, 20:1, 40:1 for 48 h. For lactic dehydrogenase (LDH) release assay, add the LDH release reagent and the supernatant was collected for absorbance measurement. For CCK8 assay, the cultural supernatant was discarded and the optical absorbance was measured after adding 10 μL CCK8 reagent for two h. When exploring the effect of different PAAG stiffness on the T cell-mediated tumor cell killing ability, we constructed the different PAAG stiffness of 96 well plates. Next, CD8 + T cells were co-cultured with DU145 and PC-3 prostate cancer cells in 96 well plates of different PAAG stiffness for 48 h.

### In vivo tumorigenesis assay

Four- to six-week-old male C57BL/6J mice were purchased from Shanghai SLAC Laboratory Animal Co., Ltd (Shanghai, China). For in vivo exploration of the biological functions of USP8, a subcutaneous model was established by subcutaneously implanting stable RM-1 cells (5 × 10^6^ cells/mouse) into the flanks of C57BL/6J mice (n = 5 mice per group). For in vivo exploration of the anti-cancer effect of USP8 inhibitor (DUBs-IN-2) and anti-PD-L1 antibody, a subcutaneous model was established by subcutaneously implanting RM-1 cells (5 × 10^6^ cells/mouse) into the flanks of C57BL/6J mice. Tumor-bearing mice were pooled and randomly divided into the following groups: (1) control; (2) USP8 inhibitor (DUBs-IN-2); (3) anti-PD-L1 antibody; (4) USP8 inhibitor in combination with anti-PD-L1 (n = 5 mice per group). All treatments were conducted by intraperitoneal injection. The anti-PD-L1 antibody was applied every 3 days and the USP8 inhibitor treatment was given with a dosage of 3 mg/kg of mouse body weight daily with a break every 6 days, as previously described. The tumors were observed and measured every three days. After 18 days, the mice were sacrificed for tumor excision, and tumor volumes were calculated using the following formula: π/6 × ab^2^ (a: length; b: width, a > b). The animal experiments were approved by the Animal Experimentation Ethics Committee of Fujian Medical University.

### Quantification of tumor-infiltrating lymphocytes in subcutaneous tumor

Tumor tissues from tumor-bearing mice were aseptically excised, minced into small pieces, and digested with DNase I and Collagenase D to obtain single-cell suspensions. The suspensions were filtered through a 70 μm strainer, and lymphocytes were isolated using density gradient centrifugation at 800 g for 30 minutes. The lymphocyte layer was collected, washed, and resuspended in PBS for downstream analysis. For surface marker staining, 10^6^ cells per sample were incubated with anti-CD45, anti-CD3, and anti-CD8 antibodies at 4 °C in the dark for 30 minutes. Cells were then washed, resuspended, and analyzed by flow cytometry. For intracellular staining, fixed and permeabilized cells were incubated with anti-GZMB and anti-PRF1 antibodies under the same conditions. After washing, cells were analyzed by flow cytometry to assess lymphocyte proportions and functional marker expression.

### Statistical analysis

All data represents at least 3 independent experiments and are shown as mean ± SD. Statistical analysis was performed with GraphPad Prism 8.0. If not mentioned otherwise in the figure legends, statistical significance (*P < 0.05; **P < 0.01; ***P < 0.001) was determined by unpaired, two-tailed Student’s t tests, two-way repeated measures ANOVA test, one-way ANOVA test, or Wilcoxon rank sum test where appropriate.

## Results

### High matrix stiffness facilitates the progression and immune evasion of PCa

**Fig. 1:**
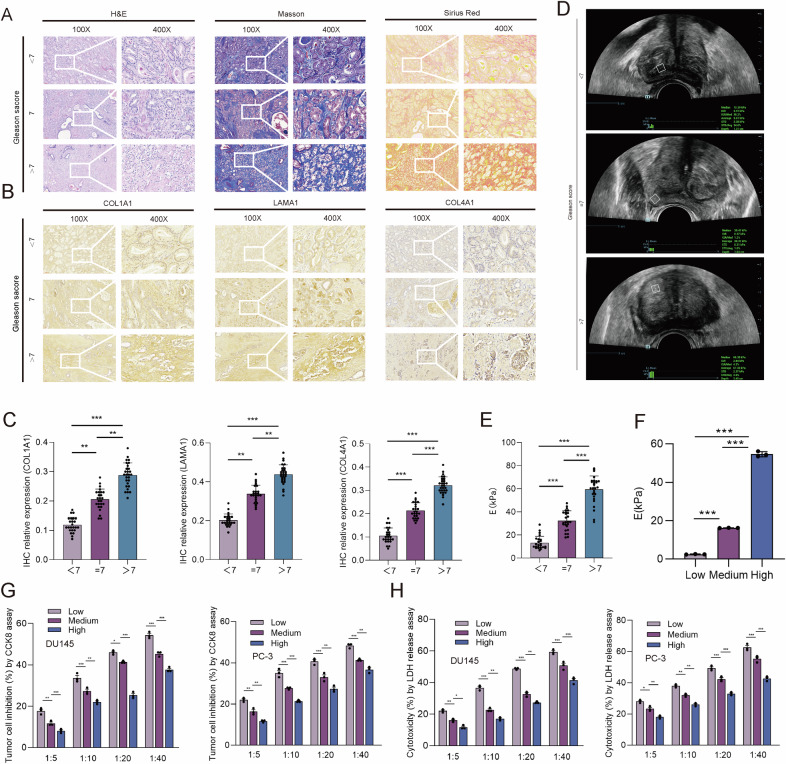
High matrix stiffness facilitates the immune evasion of PCa cells. **A** H&E staining, Masson staining, and Sirius Red staining indicating the different deposition of collagen fiber in PCa tissues of Gleason score < 7, or =7, or >7. **B** IHC staining and (**C**) the quantitative results indicating the different expression of COL1A1, LAMA1, and COL4A1 in PCa tissues of Gleason score < 7, or =7, or >7. **D** A representative image for the measure of prostate tissue stiffness via ultrasonic elastography and (**E**) the quantitative results showing the different stiffness in PCa tissues of Gleason score < 7, or =7, or >7. We used a cognitive fusion image of ultrasound, mpMRI and PSMA PET/CT to locate the region of interest (ROI), and the same ROI was measured at least 3 times and the mean value was regarded as the final value of prostate stiffness. The Gleason score was obtained from the pathological report of ROI biopsy or postoperative large pathological section of prostate. **F** The quantitative results of elastic moduli for different PAAG stiffness. **G**, **H** The effect of different PAAG stiffness on the cytotoxic T cell killing ability to DU145/PC-3 cells. Data are presented as the mean ± SD of at least three independent experiments and were analyzed with one-way ANOVA test unless otherwise stated, *P < 0.05, **P < 0.01, ***P < 0.001.

Gleason score has been proved to be closely associated with the invasiveness of PCa and the prognosis of PCa patients [12]. It has also been reported that higher Gleason score represents higher ECM mechanical stiffness, and that PCa with higher Gleason score is more susceptible to immune escape [11]. However, whether ECM mechanical stiffness is the cause of higher ability of invasiveness and immune escape of PCa with high Gleason score remains uncertain. According to prostate cancer risk stratification in the European Association of Urology (EAU) guidelines, Gleason score < 7 is considered to be a low-risk group, and Gleason score =7 is considered to be a medium-risk group, and Gleason score >7 is considered to be a high-risk group [13]. Hence, in this study, we retrospectively collected the paraffin sections of PCa patients and divided PCa patients into three sub-group: Gleason score < 7, Gleason Score=7, Gleason score > 7. H&E staining, Masson staining, and Sirius Red staining showed that the deposition of collagen fiber in the PCa tissues was elevated with the increase of Gleason score (Fig. 1A). Generally speaking, ECM was composed of basement membrane components represented by collagen type IV (COL4A1) and interstitial connective tissue components represented by collagen type I (COL1A1) and laminin (LAMA1) [14]. We conducted IHC staining in the PCa tissues of Gleason score < 7, or =7 or >7, and found that COL4A1, COL1A1, and LAMA1 was increasingly highly expressed in PCa tissues with the increase of Gleason score (Fig. 1B, C). Ultrasonic elastography has been widely used in assessing the stiffness of solid tissues, including breast, liver, prostate, and etc. [15]. We conducted the ultrasonic elastography in PCa tissues of Gleason score < 7, or =7 or >7, and found that higher Gleason score tissue presented higher stiffness (Fig. 1D, E). These results revealed that ECM stiffness was positively associated with the Gleason score of PCa, and the deposition of matrix associated proteins might be one of the vital causes of stiffness increase in PCa tissue with the increase of Gleason score.

ECM stiffening has been found to induce the carcinogenesis and increase the risk of cancer progression of breast cancer, liver cancer, and pancreas cancer [16]. To further explore whether matrix stiffness would affect the progression of PCa, we constructed the polyacrylamide hydrogels (PAAG) model with three kinds of stiffness. Strain-stress curves and calculations of elastic modulus developed by texture analyzer showed the successful construction of the PAAG model of low, medium, and high stiffness (Figs. 1F, S1). Significant changes in cell morphology and significant increase of cell spread area of DU145 and PC-3 cells were observed with the increase of PAAG stiffness (Fig. S2A). FITC-labeled phalloidine can specifically bind to the filamentous actin (F-actin) of eukaryotic cells, which can show the distribution of microfilament skeleton in the cells [17]. We conducted the phalloidine assay to reveal the changes of cytoskeleton morphology of DU145 and PC-3 cells cultured in different PAAG stiffness (Fig. S2B). The CCK8 assay, clone formation assay, and EdU staining revealed that higher PAAG stiffness could enhance the viability, colony forming capability, and proliferation ability of DU145 and PC-3 cells (Fig. S2C–E). Western blot showed that high PAAG stiffness could increase the expression of PCNA and Cyclin D1 in DU145 and PC-3 cells (Fig. S2F). Flow cytometry found that high PAAG stiffness could inhibit the apoptotic ability of DU145 and PC-3 cells (Fig. S2G). As indicated by western blot, the expressions of N-cadherin and vimentin were elevated and the expression of E-cadherin was reduced with the increase of PAAG stiffness in DU145 and PC-3 cells (Fig. S2H), revealing that high PAAG stiffness could promote the epithelial-mesenchymal transition (EMT) of DU145 and PC-3 cells. Moreover, Transwell migration and invasion assay demonstrated that high PAAG stiffness could facilitate the invasion and migration capability of DU145 and PC-3 cells (Fig. S2I, J). Besides, we co-cultured DU145/PC-3 cells with CD8 + T cells in different PAAG stiffness. The results of CD8 + T cells cytotoxicity assay revealed that the cell inhibition and cytotoxicity of CD8 + T cells to DU145 and PC-3 cells were significantly inhibited with the increase of PAAG stiffness (Fig. 1G, H). These in vitro results demonstrated that high PAAG stiffness could facilitate the progression and immune evasion of PCa cells.

### High matrix stiffness upregulates the expression of USP8 via integrin β1/FAK/YAP axis

**Fig. 2:**
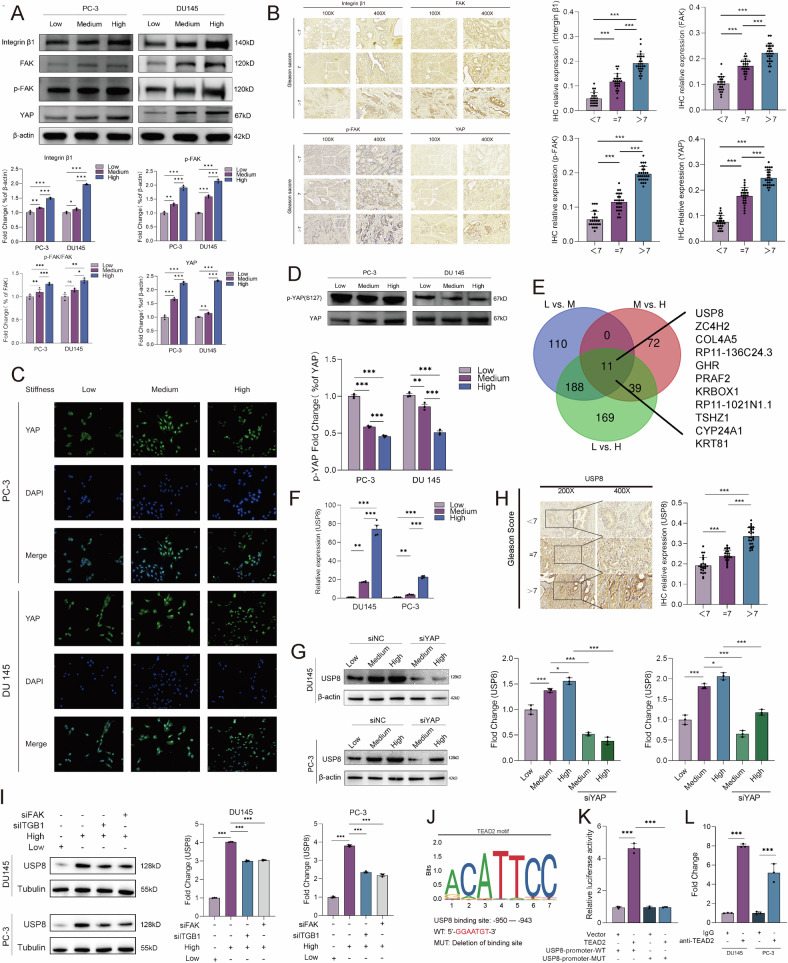
High matrix stiffness upregulates the expression of USP8 via integrin β1/FAK/YAP axis. **A** Western blot and its quantitative results indicating the relative expression of integrin β1, p-FAK, FAK, and YAP protein in DU145/PC-3 cells cultured in different PAAG stiffness. **B** IHC staining and its quantitative results indicating the different expression of integrin β1, p-FAK, FAK, and YAP protein in PCa tissues of Gleason score < 7, or =7, or >7. **C** Immunofluorescence detecting the subcellular localization of YAP protein in DU145/PC-3 cells cultured in different PAAG stiffness. **D** Western blot and relative quantification of p-YAP (S127) and YAP protein. **E** Venn diagram showing the differentially expressed genes (DEGs) detected by RNA-seq of DU145 cultured in different PAAG stiffness. **F** RT-qPCR showing the relative transcript level of USP8 in DU145/PC-3 cells cultured in different PAAG stiffness. **G** Western blot indicating the relative expression of USP8 protein in DU145/PC-3 cells cultured in different PAAG stiffness or in combination with siYAP transfection. **H** IHC staining and its quantitative results indicating the different expression of USP8 protein in PCa tissues of Gleason score < 7, or, =7, or >7. **I** Western blot indicating the relative expression of USP8 protein in DU145/PC-3 cells cultured in different PAAG stiffness or in combination with siITGB1 or siFAK transfection. **J**, **K** Relative luciferase activity in HEK-293T cells co-transfected with either TEAD2 plasmid or empty vector and either USP8-promoter-WT, or USP8-promoter-MUT vectors. **L** ChIP-qPCR was used to detect the binding of TEAD2 to the USP8 promoter in DU145 and PC-3 cells using TEAD2 primary antibody, and IgG was applied as a negative control. Data are presented as the mean ± SD of at least three independent experiments and were analyzed with unpaired two-tailed student’s t-test or one-way ANOVA test unless otherwise stated, *P < 0.05, **P < 0.01, ***P < 0.001.

ECM stiffness could activate mechanosensory / mechanoregulator proteins, for example, integrin β1, FAK, and YAP, modulating the biological behavior of tumor cells and stromal cells [18, 19]. To investigate that whether matrix stiffness affects the characteristics of PCa cells via integrin β1/FAK/YAP axis, PC-3 and DU145 cells were cultured in PAAG model of low, medium, and high stiffness, and the protein was extracted for western blot detection. The results showed that the expression of integrin β1, FAK, p-FAK, and YAP proteins was up-regulated with the increase of PAAG stiffness and the proportion of p-FAK/FAK was also positively correlated with the increase of PAAG stiffness (Fig. 2A). IHC staining also demonstrated that integrin β1, FAK, p-FAK, and YAP proteins were more highly expressed in PCa tissues of higher Gleason score (Fig. 2B). The YAP protein is also a recognized transcriptional coactivator in response to mechanical signal, and p-YAP is an inactivated form localized in cytoplasm while YAP is an activated form localized in cytoplasm cell nucleus to act as a transcriptional coactivator [20]. Immunofluorescence revealed that nuclear localization of YAP in DU145 and PC-3 cells was elevated with the increase of PAAG stiffness (Fig. 2C). Western blot found that relative proportion of p-YAP (S127)/YAP protein was significantly reduced with the increase of PAAG stiffness (Fig. 2D). These results suggested that mechanical signal could activate YAP via de-phosphorylation in S127 and promote the translocation of YAP into cell nucleus to act as a transcriptional coactivator.

To explore the key genes transcriptionally regulated by YAP, we conducted RNA-seq of DU145 cells cultured in low, medium, and high PAAG stiffness. We screened the differentially expressed genes (DEGs) by comparing the RNA-seq results between low and medium stiffness, between medium and high stiffness, between low and high stiffness (Fig. S3A–C). After taking intersection, there was a total of 11 downstream genes and we finally identified USP8 as the downstream target gene regulated by YAP (Fig. 2E). RT-qPCR and western blot confirmed that high PAAG stiffness could increase the mRNA and protein expression of USP8, which could be rescued by silencing YAP (Fig. 2F, G). IHC staining also demonstrated that the USP8 proteins were increasingly expressed in PCa tissues with the increase of Gleason score (Fig. 2H). It has been reported that YAP acts as a transcription coactivator regulating gene transcription by interacting with TEADs transcription factors family (including TEAD1, TEAD2, TEAD3 and TEAD4) after entering the nucleus [21]. We used the siRNA or overexpressed plasmid of TEAD1, TEAD2, TEAD3, and TEAD4 to transfect the PC-3 and DU145 cells. RT-qPCR showed that only TEAD2 could significantly positively affect the mRNA expression of USP8 (Fig. S3D–F). Western blot confirmed that high PAAG stiffness could increase the protein expression of USP8, which could be rescued by silencing ITGB1 or FAK (Fig. 2I). Dual-luciferase reporter assay and ChIP-qPCR assay showed that TEAD2 could promote the transcription of USP8 via binding to the promoter of USP8 (Fig. 2J–L). These results revealed that PAAG stiffness could upregulate the expression of USP8 via YAP/TEAD2-mediated transcriptional regulation.

### High PAAG stiffness promotes the progression and immune evasion of PCa via upregulating USP8

To further explore the biological function of USP8, we used lentivirus to overexpress or interfere USP8 in DU145 and PC-3 cells. RT-qPCR and western blot confirmed the efficiency of overexpression and interference of USP8 in DU145 and PC-3 cells (Fig. S4A, B). CCK8 assay and clone formation assay demonstrated that high expression of USP8 could promote the viability and colony forming capability of DU145 and PC-3 cells while low expression of USP8 could inhibit the viability and colony forming capability of DU145 and PC-3 cells (Fig. S4C, D). Transwell migration and invasion assay demonstrated that high expression of USP8 could facilitate the invasion and migration capability of DU145 and PC-3 cells while low expression of USP8 could inhibit the invasion and migration ability of DU145 and PC-3 cells (Fig. S4E, F). Besides, we co-cultured stable USP8 overexpressed or knock-down DU145/PC-3 cells with CD8 + T cells. The results of CD8 + T cells cytotoxicity assay revealed that high expression of USP8 could promote the immune evasion of DU145 and PC-3 PCa cells while low expression of USP8 could inhibit the immune evasion of DU145 and PC-3 PCa cells (Fig. S4G, H). Stable USP8-overexpressed RM-1 cells and control cells, stable USP8-silenced RM-1 cells and control cells were subcutaneously transplanted into C57BL/6J mice and investigate the impact of USP8 expression level in tumor growth in vivo. Tumor growth in the sh-USP8 group was significantly slower than that in the control group, and tumor growth in the USP8 overexpression group was significantly faster than that in the control group. The tumor weights and volumes were smaller in the sh-USP8 group than that in the control group, and the tumor weights and volumes were increased in the USP8 overexpression group than that in the control group (Fig. S4I–K). Flow cytometry was used to quantitatively analyze the proportion of CD3^+^ T lymphocytes in CD45^+^ cells, CD8^+^ T lymphocytes in CD45^+^CD3^+^ cells, and the proportion of GZMB^+^ cells or PRF1^+^ cells in CD8^+^ T lymphocytes. The results showed that the proportion of CD3^+^ T lymphocytes in CD45^+^ cells, CD8^+^ T lymphocytes in CD45^+^CD3^+^ cells, and the proportion of GZMB^+^ cells or PRF1^+^ cells in CD8^+^ T lymphocytes were increased after USP8 silencing and were decreased after USP8 overexpression (Fig. S4L). These results showed that USP8 could promote the progression and immune evasion of PCa.

**Fig. 3:**
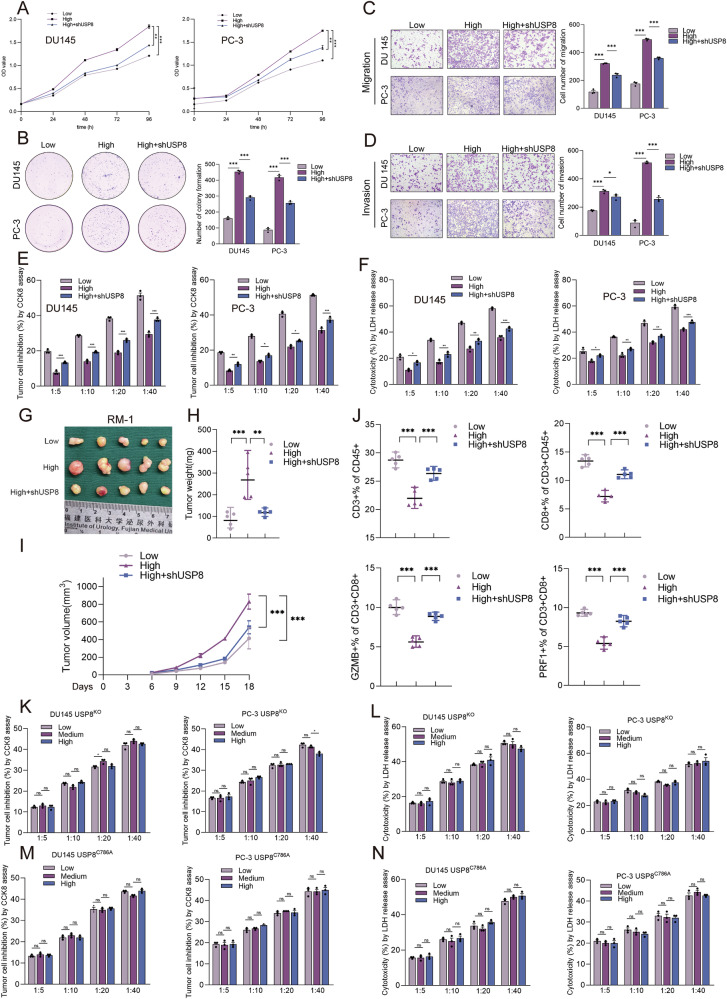
High matrix stiffness promotes the progression and immune evasion of PCa via upregulating USP8. **A** CCK8 assay showing the cell viability of DU145/PC-3 cells cultured in different PAAG stiffness or in combination with USP8 shRNA transduction. **B** Representative images and quantitative results of colony formation assay of DU145/PC-3 cells cultured in different PAAG stiffness or in combination with USP8 shRNA transduction. **C**, **D** Representative images and quantitative results of Transwell assay of DU145/PC-3 cells cultured in different PAAG stiffness or in combination with USP8 shRNA transduction. **E**, **F** The cytotoxic T cell killing ability to DU145/PC-3 cells cultured in different PAAG stiffness or in combination with USP8 shRNA transduction. **G**–**I** RM-1 cells, cultured in different PAAG stiffness or in combination with USP8 shRNA transduction, were injected into the flank of mice. Tumor volumes were measured every 3 days. Tumor images, weight and growth curves were obtained at day 18 after dissection. **J** Flow cytometry was used to quantitatively analyze the proportion of CD3^+^ T lymphocytes in CD45^+^ cells, CD8^+^ T lymphocytes in CD45^+^CD3^+^ cells, and the proportion of GZMB^+^ cells or PRF1^+^ cells in CD8^+^ T lymphocytes in the subcutaneous tumor samples of mice. The X axis represents the different group. **K**, **L** USP8^KO^ DU145/PC-3 cells were constructed and the effect of PAAG stiffness on the cytotoxic T cell killing ability to USP8^KO^ DU145/PC-3 cells was explored. **M**, **N** We transfected USP8 C786A (dead USP8) plasmid into USP8^KO^ DU145/PC3 cells. The effect of PAAG stiffness on the cytotoxic T cell killing ability to USP8^C786A^ DU145/PC-3 cells was explored. Data in A and I were analyzed by two-way repeated measures ANOVA test. Data are presented as the mean ± SD of at least three independent experiments and were analyzed with unpaired two-tailed student’s t-test or one-way ANOVA test unless otherwise stated, *P < 0.05, **P < 0.01, ***P < 0.001.

Next, we explored whether high PAAG stiffness affected the progression of PCa cells via upregulating the expression of USP8. CCK8 assay and clone formation assay demonstrated that USP8-knockdown significantly inhibits the cell viability and colony forming capability of DU145 and PC-3 cells cultured in high PAAG stiffness (Fig. 3A, B). Transwell migration and invasion assay revealed that USP8-knockdown significantly inhibits the invasion and migration capability of DU145 and PC-3 cells cultured in high PAAG stiffness (Fig. 3C, D). Besides, we co-cultured stable DU145/PC-3 cells with CD8 + T cells. The results of CD8 + T cells cytotoxicity assay revealed that the silencing of USP8 could rescue the promoting role of high PAAG stiffness on the immune evasion of DU145 and PC-3 PCa cells (Fig. 3E, F). Next, the assay of subcutaneous tumors formed by RM-1 cells were also conducted. Tumor growth in the high PAAG stiffness group was significantly faster than that in the low PAAG stiffness group, and could be rescue by USP8 silencing. The tumor weights and volumes were increased in the high PAAG stiffness group than that in the low PAAG stiffness group, and could be rescue by USP8 silencing (Fig. 3G–I). Flow cytometry was used to quantitatively analyze the proportion of CD3^+^ T lymphocytes in CD45^+^ cells, CD8^+^ T lymphocytes in CD45^+^CD3^+^ cells, and the proportion of GZMB^+^ cells or PRF1^+^ cells in CD8^+^ T lymphocytes. The results showed that the proportion of CD3^+^ T lymphocytes in CD45^+^ cells, CD8^+^ T lymphocytes in CD45^+^CD3^+^ cells, and the proportion of GZMB^+^ cells or PRF1^+^ cells in CD8^+^ T lymphocytes were decreased in the high PAAG stiffness group than that in the low PAAG stiffness group, and could be rescued by USP8 silencing (Fig. 3J). Moreover, we constructed USP8^KO^ DU145/PC-3 cell lines, and then transfected USP8 C786A (dead USP8) plasmid into USP8^KO^ DU145/PC3 cell lines. It was found that the effect of PAAG stiffness on the CD8^+^ T cells cytotoxicity to USP8^KO^ DU145/PC-3 cells and USP8^C786A^ DU145/PC-3 cells was lost, indicating the vital role of USP8 and its deubiquitinating enzyme activity in the process of matrix stiffness promoting immune escape of PCa (Fig. 3K–N). The results showed that PAAG stiffness promoted the proliferation, invasion, migration, and immune escape of PCa cells via upregulating the expression level of USP8.

### USP8 specifically interacts with and stabilizes autophagy cargo receptor NBR1 protein via K63 de-ubiquitination

**Fig. 4:**
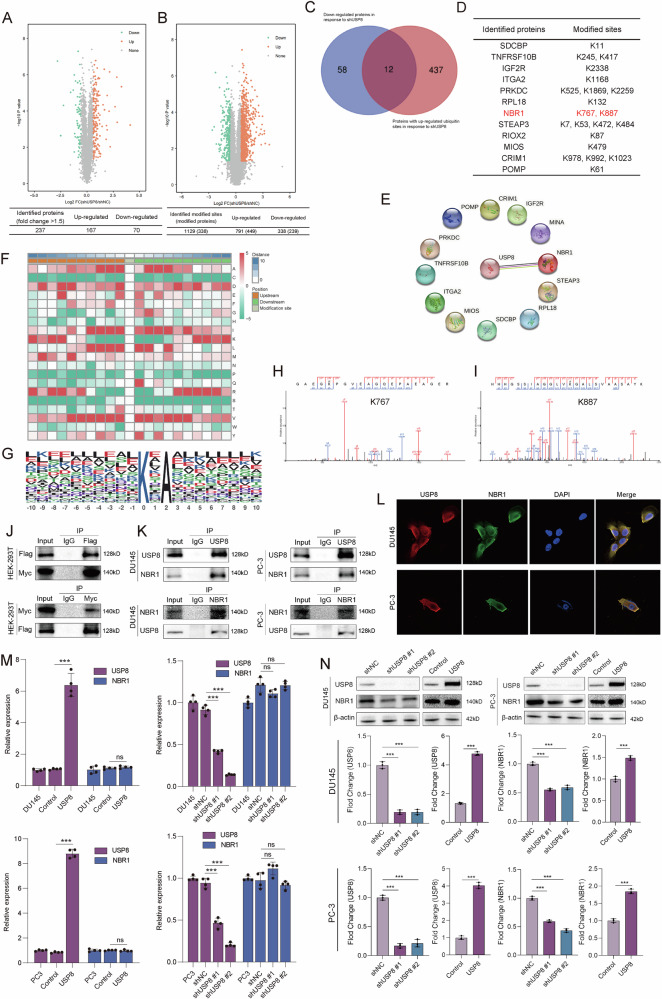
USP8 specifically interacts with autophagy cargo receptor NBR1. **A** Volcano plot showing the differentially expressed protein detected by 4D label free quantitative proteomics of DU145 cells transduced with USP8 shRNA or shNC. **B** Volcano plot showing the differentially ubiquitinated protein detected by 4D label free quantitative ubiquitinome of DU145 cells transduced with USP8 shRNA or shNC. **C** Venn diagram showing the number of down-regulated proteins and ubiquitinated proteins with up-regulated ubiquitin sites in response to shUSP8. **D** The list of 12 expressed down-regulated protein with up-regulated ubiquitin sites and the corresponding ubiquitin sites. **E** STRING database indicating the interaction of USP8 and 12 down-regulated protein with up-regulated ubiquitin sites. **F**, **G** Ubiquitination motifs consisting of 20 residues surrounding the modified lysine site analyzed by Motif-x. **H**, **I** The possible ubiquitin sites of NBR1 through mass spectrometry analysis. **J** The exogenous interaction between Flag-USP8 and Myc-NBR1 was detected in HEK-293T cells by performing coimmunoprecipitation and western blot. **K** The endogenous interaction between USP8 and NBR1 was detected in DU145 and PC-3 cells by coimmunoprecipitation and western blot. **L**Co-localization of USP8 and NBR1 was visualized in DU145 and PC-3 cells via immunofluorescence. **M** RT-qPCR assay showing the effect of USP8 knockdown or overexpression on the expression levels of NBR1 mRNA. **N**Western blot showing the effect of USP8 knockdown or overexpression on the expression levels of NBR1 protein. Data are presented as the mean ± SD of at least three independent experiments and were analyzed with unpaired two-tailed student’s t-test or one-way ANOVA test unless otherwise stated, ***P < 0.001.

USP8 belongs to the ubiquitin-specific processing protease family of proteins. To investigate the specifically targeted de-ubiquitinating substrate of USP8, we performed 4D label free quantitative proteomics and ubiquitinome in stable USP8-silenced DU145 cells and control cells. Quantitative proteomics identified a total of 167 upregulated proteins and 70 downregulated proteins (Fig. 4A). After being normalized by quantitative proteomics, quantitative ubiquitinome identified a total of 791 upregulated ubiquitin modified sites in 449 proteins and 338 downregulated ubiquitin modified sites in 239 proteins (Fig. 4B). For the reason that USP8 plays an important regulatory role in removing conjugated ubiquitin from proteins, therefore preventing the degradation of protein, we took the intersections of down-regulated proteins and ubiquitinated proteins with up-regulated ubiquitin sites in response to shUSP8 (Fig. 4C). The list of a total of 12 intersecting down-regulated protein with up-regulated ubiquitin sites and the corresponding ubiquitin sites was presented in Fig. 4D. We imported these 12 proteins and USP8 in STRING database, and found that only NBR1 exists a potential interaction with USP8 (Fig. 4E) Ubiquitination motifs consisting of 20 residues surrounding the modified lysine site by Motif-x was showed in Fig. 4F, G and the peak map of the possible ubiquitin sites of NBR1 (K767 and K887) identified by mass spectrometry analysis was presented in Fig. 4H–I.

However, there was no study reporting that NBR1 could be ubiquitinated and then degraded. We conducted endogenous in vivo ubiquitination assay and found that NBR1 could be ubiquitinated in PC-3 and DU145 cells (Fig. S5A). Autophagy-lysosome pathway and ubiquitin proteasome pathway were two major ubiquitin-dependent degradation pathway of proteins [22]. We found that pretreatment with the lysosome inhibitor 3-MA increased NBR1 protein level in DU145 and PC-3 cells, whereas the proteasome inhibitor MG132 failed to affect NBR1 protein expression (Fig. S5B), indicating that NBR1 might be degraded through autophagy-lysosome pathway. To explore the E3 ubiquitin ligase that mediates NBR1 ubiquitination modification and the autophagy cargo receptor that mediates NBR1 degradation, the protein products were analyzed using mass spectrometry after immunoprecipitation. Several E3 ubiquitin ligase, including RBBP6, MIB1, HERC2, TRIM21, UBR5, were identified as potential interacting partners of NBR1, but only TRIM21 was confirmed by Co-immunoprecipitation in DU145 cells (Fig. S5C, D). Endogenous in vivo ubiquitination assay revealed that the polyubiquitination level of NBR1 was markedly decreased after interference of TRIM21 in PC-3 and DU145 cells (Fig. S5E). Several autophagy cargo receptors, including P62/SQSTM1 and TAX1BP1, were identified as potential interacting partners of NBR1, but only P62/SQSTM1 was confirmed by Co-immunoprecipitation in DU145 cells (Fig. S5F, G). These results demonstrated that NBR1 could be ubiquitinated and then degraded via interacting with P62/SQSTM1 and through autophagy-lysosome pathway.

Then, to verify the results of proteomics and ubiquitinome, we co-transfected Flag-USP8 and Myc-NBR1 into HEK-293T cells. The results of Co-IP and western blot revealed that there was an exogenous specifical interaction between Flag-USP8 and Myc-NBR1 (Fig. 4J). The endogenous interaction between USP8 and NBR1 was also confirmed by Co-IP and immunoblotting assays in DU145 and PC-3 cells (Fig. 4K). The immunofluorescence demonstrated the co-localization of USP8 protein and NBR1 protein (Fig. 4L). Furthermore, the overexpression or knockdown of USP8 in DU145 and PC-3 cells failed to affect the expression level of NBR1 mRNA (Fig. 4M); however, the silence of USP8 in DU145 and PC-3 cells could significantly reduce the level of NBR1 protein and the overexpression of USP8 in DU145 and PC-3 cells could significantly elevate the level of NBR1 protein (Fig. 4N). These results suggested that USP8 specifically interacted with NBR1 protein and affected the expression of NBR1 protein but not mRNA.

**Fig. 5:**
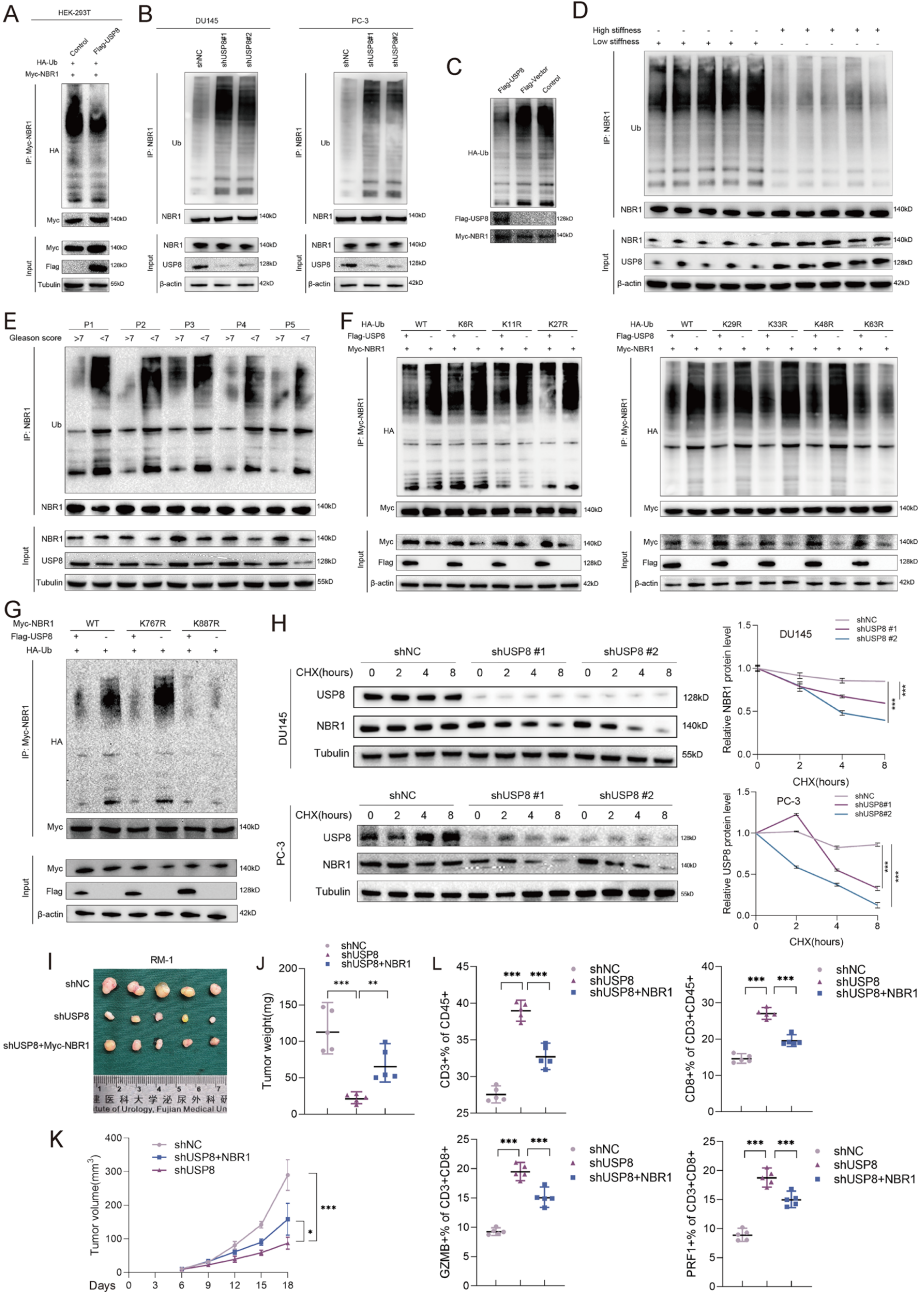
USP8 deubiquitinates and stabilizes NBR1. **A** Exogenous ubiquitination assay of Myc-NBR1 in HEK-293T cells transduced with Flag-USP8 together with Myc-NBR1 and HA-Ub plasmid, and treated with 3-MA for 6 h. **B** Endogenous ubiquitination assay of NBR1 in DU145 and PC-3 cells treated with 3-MA for 6 h. **C** In vitro de-ubiquitination assay of ubiquitinated Myc-NBR1 protein with purified Flag-USP8. **D** Ubiquitination assay of NBR1 in subcutaneous tumors derived from RM-1 cells in high or low PAAG stiffness. **E** Ubiquitination assay of NBR1 in PCa tissues of Gleason score < 7 or >7. **F** Ubiquitination assay of Myc-NBR1 in HEK-293T cells co-transfected with Myc-NBR1, Flag-USP8 together with HA-Ub, HA-Ub-K6R, HA-Ub-K11R, HA-Ub-K27R, HA-Ub-K29R, HA-Ub-K33R, HA-Ub-K48R, HA-Ub-K63R, and treated with 3-MA for 6 h. **G** Ubiquitination assay of Myc-NBR1 in HEK-293T cells co-transfected with HA-Ub, Flag-USP8 together with Myc-NBR1, Myc-NBR1-K767R, Myc-NBR1-K887R, and treated with 3-MA for 6 h. **H** Stability analysis of NBR1 protein in DU145 and PC-3 cells transfected with shNC or shUSP8, and treated with 40 μM cycloheximide (CHX) for indicated times. **I**–**K** RM-1 related stable cells (shNC, shUSP8, and shUSP8+Myc-NBR1) were injected into the flanks of mice, respectively. Tumor volumes were measured every 3 days. Tumor images, growth curves and weight were obtained at day 18 after dissection. **L** Flow cytometry was used to quantitatively analyze the proportion of CD3^+^ T lymphocytes in CD45^+^ cells, CD8^+^ T lymphocytes in CD45^+^CD3^+^ cells, and the proportion of GZMB^+^ cells or PRF1^+^ cells in CD8^+^ T lymphocytes. The X axis represents the different group. Data in H and K were analyzed by two-way repeated measures ANOVA test. Data are presented as the mean ± SD of at least three independent experiments and were analyzed with one-way ANOVA test unless otherwise stated, *P < 0.05, **P < 0.01, ***P < 0.001.

Next, we explore whether USP8 could remove the conjugated ubiquitin from NBR1 protein and regulate the stability of the NBR1 protein. Then, we performed ubiquitination assay to investigate the de-ubiquitinated role of USP8 in NBR1 protein. Exogenous in vivo ubiquitination assay revealed that the polyubiquitination level of Myc-NBR1 was markedly decreased in Flag-USP8 transduced HEK-293T cells (Fig. 5A). Endogenous in vivo ubiquitination assay showed that the polyubiquitination level of NBR1 was markedly increased in stable USP8-silenced DU145/PC-3 cells (Fig. 5B). Furthermore, USP8 could also significantly promote the in vitro NBR1 de-ubiquitination (Fig. 5C). Tumors formed by RM-1 cells in high PAAG stiffness exhibited downregulation of NBR1 polyubiquitination compared to tumors formed by RM-1 cells in low PAAG stiffness, and could be rescued by the knockdown of USP8 (Figs. 5D and S6A). In addition, the polyubiquitination of NBR1 was also inhibited in high Gleason score (>7) tissues compared with that in low Gleason score (<7) tissues (Fig. 5E). Ubiquitin is a protein composed of 76 amino acids with seven lysine residues (K6, K11, K27, K29, K33, K48, and K63), all of which can be ubiquitinated to form a unique polyubiquitin chain. To identify which lysine residues of ubiquitin are involved in USP8 de-ubiquitination process of NBR1, we mutated K6, K11, K27, K29, K33, K48, K63 lysine residues on the ubiquitin to arginine (K6R, K11R, K27R, K29R, K33R, K48R, K63R) and co-transfected Myc-NBR1, Flag-USP8, and HA-Ub (including wide type and mutated type) into HEK-293T cells. The results showed that it was K63 lysine residue on polyubiquitin chain involved in the USP8-medicated de-ubiquitination of NBR1 (Fig. 5F). Endogenous ubiquitination assay using K63-linkage or K48-linkage specific polyubiquitin antibody further confirmed the role of K63 lysine residue in the USP8-medicated de-ubiquitination of NBR1 (Fig. S6B). To identify whether K767 (Lys-767) and K887 (Lys-887) lysine sites on the NBR1 identified by ubiquitinome are involved in USP8-mediated de-ubiquitination process, we mutated K767 and K887 lysine to arginine on the NBR1 and co-transfected Myc-NBR1 (including wide type and K767R-mutation and K887R-mutation), Flag-USP8, and HA-Ub into HEK-293T cells. The results showed that the Lys-887 rather than Lys-767 was an important site for USP8-medicated de-ubiquitination of NBR1 (Fig. 5G). Notably, we found that knockdown of USP8 led to a prominent decrease in the stability of endogenous NBR1 protein in DU145 and PC-3 cells, whereas the stability of tubulin was not affected (Fig. 5H).

### USP8 enhances the progression and immune evasion of PCa via upregulating NBR1

To determine whether USP8 promotes the progression of PCa via upregulating NBR1, we modified the expression of NBR1 in USP8-knockdown cells. NBR1-transduction in the USP8-silencing DU145 and PC-3 cells significantly increased the cell viability, colony forming capability, invasion and migration capability of USP8-silencing PCa cells (Fig. S7A–D). Besides, we co-cultured stable DU145/PC-3 cells with CD8 + T cells. The results of CD8 + T cells cytotoxicity assay revealed that the inhibiting role of USP8 silence on the CD8 + T cells killing ability to DU145 and PC-3 cells could be rescued by NBR1 overexpression (Fig. S7E, F). Mice implanted with RM-1 cells with simultaneous USP8 knockdown and NBR1 overexpression showed higher tumor growth rates compared with those bearing USP8-silencing cells (Fig. 5I, K). Flow cytometry was used to quantitatively analyze the proportion of CD3^+^ T lymphocytes in CD45^+^ cells, CD8^+^ T lymphocytes in CD45^+^CD3^+^ cells, and the proportion of GZMB^+^ cells or PRF1^+^ cells in CD8^+^ T lymphocytes. The results showed that the proportion of CD3^+^ T lymphocytes in CD45^+^ cells, CD8^+^ T lymphocytes in CD45^+^CD3^+^ cells, and the proportion of GZMB^+^ cells or PRF1^+^ cells in CD8^+^ T lymphocytes were increased after USP8 silencing and could be rescued by NBR1 overexpression (Fig. 5L). Collectively, these results revealed that USP8 enhanced the progression and immune evasion of PCa via upregulating NBR1.

### High PAAG stiffness reduces the expression levels of MHC-1 via USP8/NBR1 axis

**Fig. 6:**
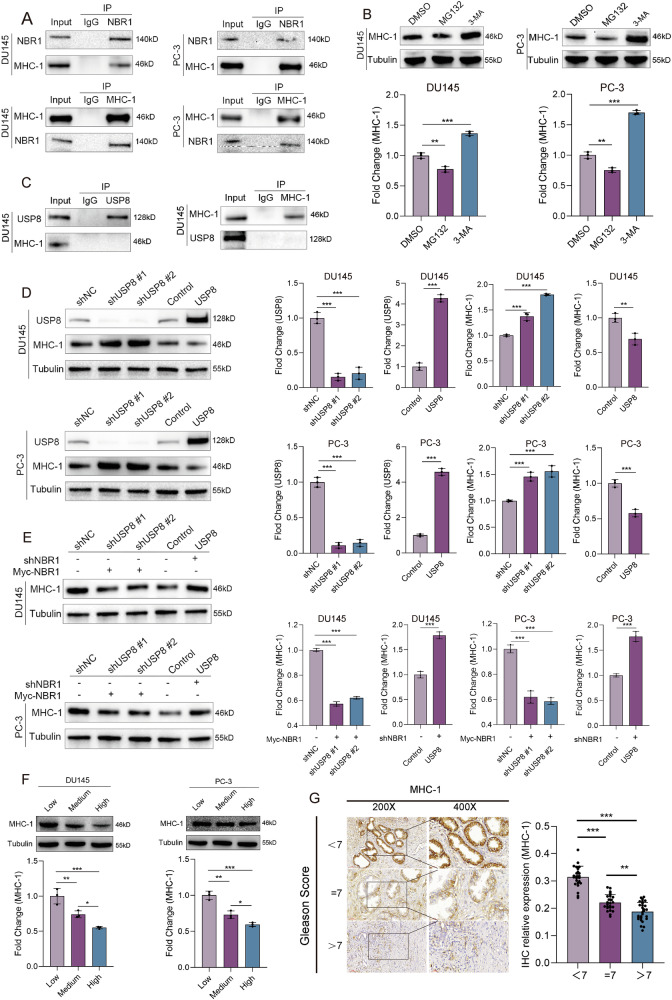
High matrix stiffness downregulates MHC-I expression through USP8/NBR1 axis. **A** The endogenous interaction between MHC-1 and NBR1 was detected in DU145 and PC-3 cells by coimmunoprecipitation and western blot. **B** Western blot analysis of MHC-1 expression in DU145 and PC-3 cells treated with 3-MA or MG132. **C** Coimmunoprecipitation and western blot indicating the endogenous non-interaction between USP8 and MHC-1 in DU145 cells. **D** Western blot showing the effect of USP8 knockdown or overexpression on the expression levels of MHC-1 protein in DU145 and PC-3 cells. **E** Western blot indicating that the effect of USP8 on MHC-1 expression could be rescued by NBR1. **F** Western blot and its quantitative results indicating the expression levels of MHC-1 of DU145 and PC-3 cultured in different PAAG stiffness. **G** IHC staining and its quantitative results indicating the different expression of MHC-1 protein in PCa tissues of Gleason score < 7, or =7, or >7. Data are presented as the mean ± SD of at least three independent experiments and were analyzed with unpaired two-tailed student’s t-test or one-way ANOVA test unless otherwise stated, *P < 0.05, **P < 0.01, ***P < 0.001.

MHC-I is essential for presentation of endogenous antigen by tumor cells and subsequent recognition and clearance by cytotoxic T lymphocytes [23]. It has been reported that MHC-1 molecule was degraded through NBR1-mediated selective autophagy in glioma and pancreatic cancer, which was considered as the major cause of immune escape [24, 25]. Besides, ECM stiffness has been found to modulate the phenotype of infiltrating T lymphocytes [26] and induce immune evasion of cancer cells [27]. Our results also showed that NBR1 binds to MHC-1 in DU145 and PC-3 cells (Fig. 6A). Besides, we also found that pretreatment with the lysosome inhibitor 3-MA significantly increased the levels of MHC-1 (Fig. 6B), suggesting that MHC-1 was also degraded by NBR1-mediated selective autophagy in PCa. However, USP8 showed no endogenous interaction with MHC-1 although knockdown of USP8 could increase the level of MHC-1 and overexpression of USP8 could reduce the level of MHC-1 (Fig. 6C, D). To determine whether USP8 affect the level of MHC-1 via NBR1, we either silenced NBR1 in USP8-overexpressing DU145/PC-3 cells or transduced NBR1 plasmid in USP8-knockdown DU145/PC-3 cells. The results revealed that NBR1 silencing could elevate the level of MHC-1 in USP8-overexpressing DU145/PC-3 cells and NBR1 transduction could reduce the level of MHC-1 in USP8-knockdown cells (Fig. 6E). Besides, the level of MHC-1 was also decreasingly expressed in DU145/PC-3 cells with the increase of PAAG stiffness (Fig. 6F) and could be rescued by autophagy inhibitor (3-MA), or the silencing of USP8 or NBR1. If we overexpressed USP8 in NBR1-silenceing PCa cells cultured in high PAAG stiffness, MHC-1 was down-regulated. However, if NBR1-silenceing PCa cells cultured in high PAAG stiffness was treated with autophagy activator (rapamycin), MHC-1 was further up-regulated. If we overexpressed wild-type NBR1 in PCa cells cultured in low PAAG stiffness, MHC-1 was down-regulated, and if we overexpressed K887R-NBR1 in PCa cells cultured in low PAAG stiffness, MHC-1 was further down-regulated compared with those transfected with wild-type NBR1 (Fig. S8). More importantly, the IHC staining revealed that MHC-1 was decreasingly expressed in PCa tissue with the increase of Gleason score (Fig. 6G). These results demonstrated that high PAAG stiffness promoted the degradation of MHC-1 and facilitate the immune evasion via USP8/NBR1 mediated selective autophagy in PCa.

### USP8 specifically interacts with and stabilizes PD-L1 protein via K48 de-ubiquitination

**Fig. 7:**
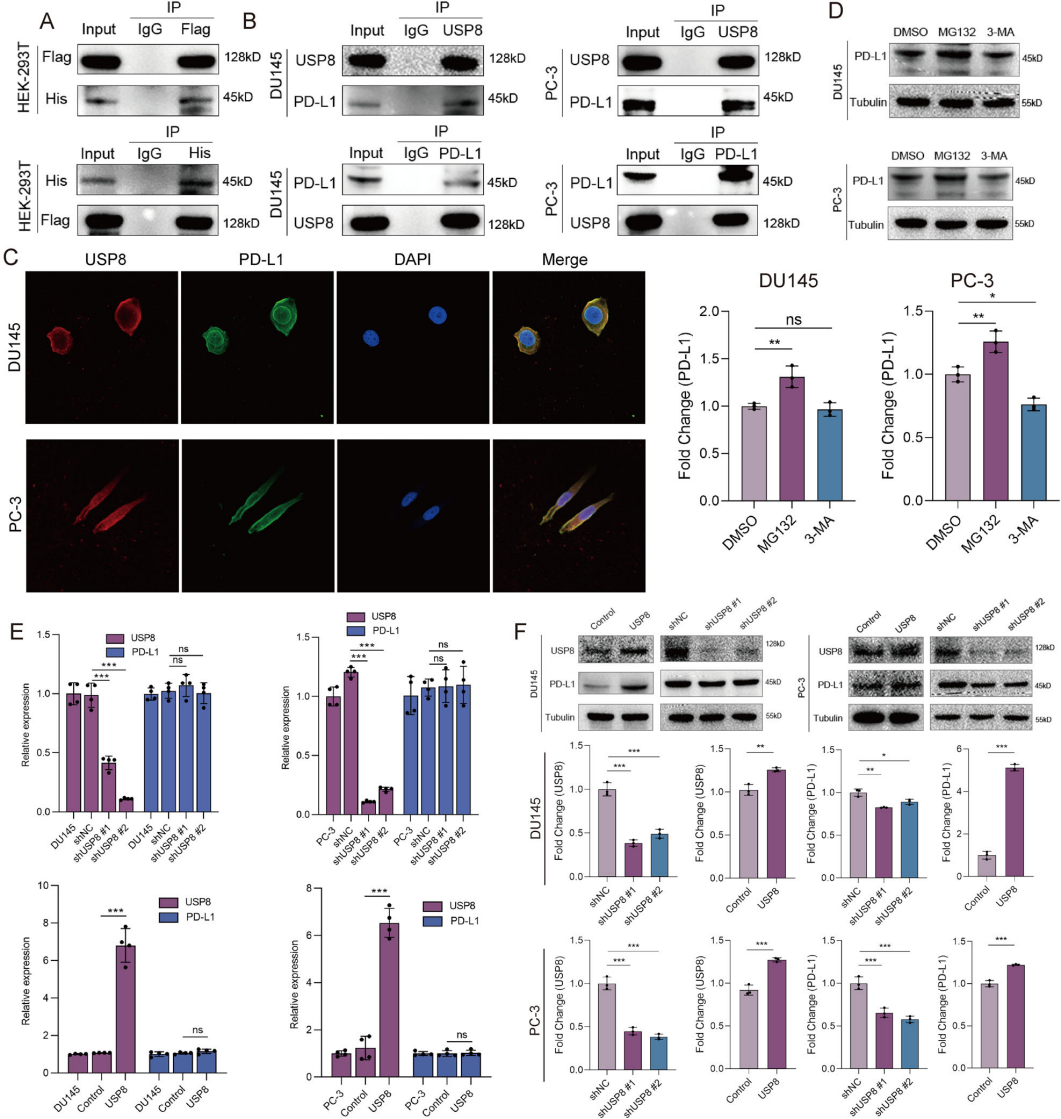
USP8 specifically interacts with PD-L1. **A** The exogenous interaction between Flag-USP8 and His-PD-L1 was detected in HEK-293T cells by performing coimmunoprecipitation and western blot. **B** The endogenous interaction between USP8 and PD-L1 was detected in DU145 and PC-3 cells by coimmunoprecipitation and western blot. **C** Co-localization of USP8 and PD-L1 was visualized in DU145 and PC-3 cells via immunofluorescence. **D** Western blot analysis of PD-L1 expression in DU145 and PC-3 cells treated with 3-MA or MG132. **E** RT-qPCR assay showing the effect of USP8 knockdown or overexpression on the expression levels of PD-L1 mRNA. **F** Western blot showing the effect of USP8 knockdown or overexpression on the expression levels of PD-L1 protein. Data are presented as the mean ± SD of at least three independent experiments and were analyzed with unpaired two-tailed student’s t-test or one-way ANOVA test unless otherwise stated, *P < 0.05, **P < 0.01, ***P < 0.001.

The increased abundance of PD-L1 in PCa cells has also considered as one of the major causes of immune evasion of PCa [28], and it has been reported that PD-L1 is an downstream deubiquitinating substrate of USP8 in pancreatic cancer [22] and non-small cell lung cancer [29]. However, whether USP8 could de-ubiquitinate and stabilize PD-L1 in PCa remains uncertain. To address this problem, we co-transfected Flag-USP8 and His-PD-L1 into HEK-293T cells. The results of Co-IP and western blot revealed that there was an exogenous specifical interaction between Flag-USP8 and His-PD-L1 (Fig. 7A). The endogenous interaction between USP8 and PD-L1 was also confirmed by Co-IP and immunoblotting assays in DU145 and PC-3 cells (Fig. 7B). The immunofluorescence demonstrated the co-localization of USP8 protein and PD-L1 protein (Fig. 7C). We found that pretreatment with the proteasome inhibitor MG132 increased PD-L1 protein level in PCa cells, whereas the lysosome inhibitor 3-MA failed to affect PD-L1 protein expression (Fig. 7D), suggesting that PD-L1 was degraded through ubiquitin-proteasome pathway in PCa. Furthermore, the overexpression or knockdown of USP8 in DU145 and PC-3 cells could not affect the expression level of PD-L1 mRNA (Fig. 7E); however, the silence of USP8 in DU145 and PC-3 cells could significantly reduce the level of PD-L1 protein and the overexpression of USP8 in DU145 and PC-3 cells could significantly elevate the level of PD-L1 protein (Fig. 7F). These results revealed that USP8 specifically interacts with PD-L1 protein and affects the expression level of PD-L1 protein but not mRNA in PCa.

**Fig. 8:**
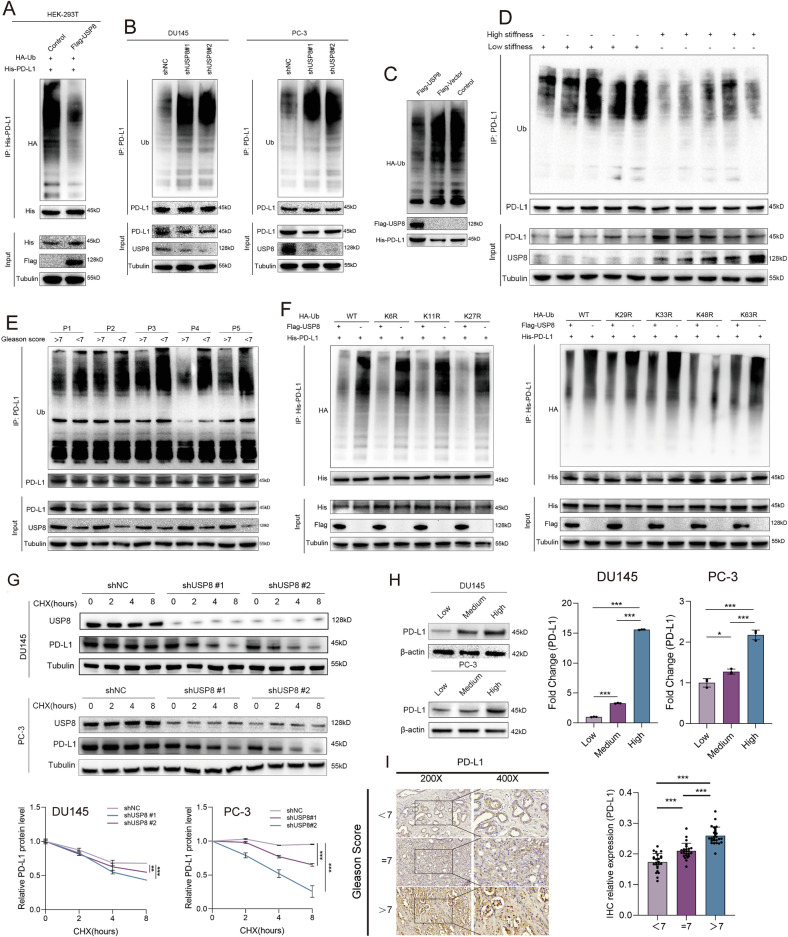
USP8 deubiquitinates and stabilizes PD-L1. **A** Exogenous ubiquitination assay of His-PD-L1 in HEK-293T cells transduced with Flag-USP8 together with His-PD-L1 and HA-Ub, and treated with MG132 for 6 h. **B** Endogenous ubiquitination assay of PD-L1 in DU145 and PC-3 cells treated with MG132 for 6 h. **C** In vitro de-ubiquitination assay of ubiquitinated His-PD-L1 protein with purified Flag-USP8. **D** Ubiquitination assay of PD-L1 in subcutaneous tumors derived from RM-1 cells in high or low PAAG stiffness. **E** Ubiquitination assay of PD-L1 in PCa tissues of Gleason score < 7 or >7. **F** Ubiquitination assay of His-PD-L1 in HEK-293T cells co-transfected with His-PD-L1, Flag-USP8 together with HA-Ub, HA-Ub-K6R, HA-Ub-K11R, HA-Ub-K27R, HA-Ub-K29R, HA-Ub-K33R, HA-Ub-K48R, HA-Ub-K63R, and treated with MG132 for 6 h. **G** Stability analysis of PD-L1 protein in DU145 and PC-3 cells transfected with shNC or shUSP8, and treated with 40 μM cycloheximide (CHX) for indicated times. **H** Western blot indicating the relative expression of PD-L1 protein in DU145 and PC-3 cells cultured in different PAAG stiffness. **I** IHC staining and its quantitative results indicating the different expression of PD-L1 protein in PCa tissues of Gleason score < 7, or =7, or >7. Data in G were analyzed by two-way repeated measures ANOVA test. Data are presented as the mean ± SD of at least three independent experiments and were analyzed with unpaired two-tailed student’s t-test or one-way ANOVA test unless otherwise stated, *P < 0.05, **P < 0.01, ***P < 0.001.

Next, we performed ubiquitination assay to investigate the de-ubiquitinated role of USP8 on PD-L1 protein. Exogenous in vivo ubiquitination assay revealed that the polyubiquitination level of His-PD-L1 was obviously decreased in Flag-USP8 transduced HEK-293T cells (Fig. 8A). Endogenous in vivo ubiquitination assay also demonstrated that the polyubiquitination level of PD-L1 was obviously increased in stable USP8-silenced DU145/PC-3 cells (Fig. 8B). Furthermore, USP8 significantly promoted the in vitro PD-L1 de-ubiquitination (Fig. 8C). Tumors formed by RM-1 cells in high PAAG stiffness exhibited downregulation of PD-L1 polyubiquitination compared to tumors formed by RM-1 cells in low PAAG stiffness, and could be rescued by the knockdown of USP8 (Figs. 8D and S9A). Moreover, polyubiquitination of PD-L1 was suppressed in high Gleason score (>7) tissues compared with low Gleason score (<7) tissues (Fig. 8E). Besides, we mutated K6, K11, K27, K29, K33, K48, K63 lysine residues on the ubiquitin to arginine (K6R, K11R, K27R, K29R, K33R, K48R, K63R) and co-transfected His-PD-L1, Flag-USP8, and HA-Ub (including wide type and mutated type) into HEK-293T cells. The results showed that it was K48 lysine residue on polyubiquitin chain involved in the USP8-mediated de-ubiquitination of PD-L1 (Fig. 8F). Endogenous ubiquitination assay using K48-linkage or K63-linkage specific polyubiquitin antibody further confirmed the role of K48 lysine residue in the USP8-mediated de-ubiquitination of PD-L1 in DU145 and PC-3 cells (Fig. S9B). Besides, knockdown of USP8 resulted in a prominent decrease in the stability of endogenous PD-L1 protein in DU145 and PC-3 cells (Fig. 8G). The level of PD-L1 was also increasingly expressed in DU145/PC-3 cells with the increase of PAAG stiffness (Fig. 8H). More importantly, the IHC staining revealed that PD-L1 was increasingly expressed in PCa tissue with the increase of Gleason score (Fig. 8I). These results revealed that high PAAG stiffness promotes the stabilization of PD-L1 protein via USP8-mediated K48-de-ubiquitination in PCa.

### Extracellular matrix-derived mechanical stiffness promotes the progression and immune evasion of PCa via USP8-mediated MHC-1 degradation and PD-L1 abundance

**Fig. 9:**
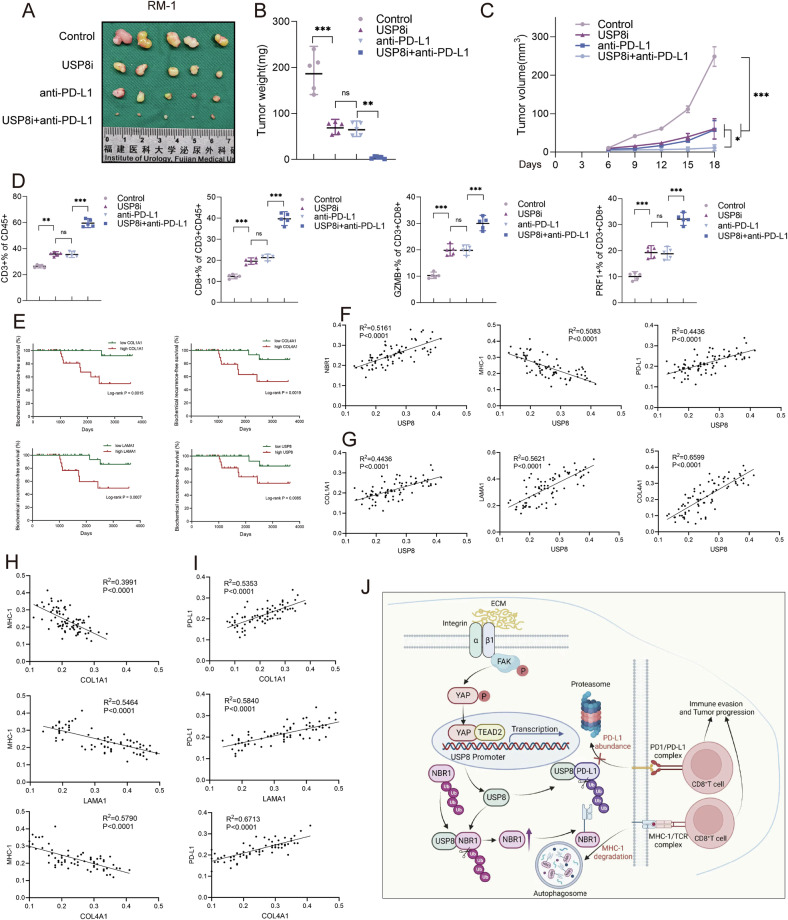
Exploration of USP8 inhibitor/anti-PD-L1 combination therapy and clinical sample validation of molecular mechanism. **A**–**C** RM-1 cells were injected into the flanks of C57BL/6J mice and randomly divided into four group: the untreated control group, the USP8 inhibitor group, the anti-PD-L1 therapy group, the USP8 inhibitor and anti-PD-L1 combination therapy group. Tumor volumes were measured every 3 days. Tumor images, growth curves and weight were obtained at day 18 after dissection. **D** Flow cytometry was used to quantitatively analyze the proportion of CD3^+^ T lymphocytes in CD45^+^ cells, CD8^+^ T lymphocytes in CD45^+^CD3^+^ cells, and the proportion of GZMB^+^ cells or PRF1^+^ cells in CD8^+^ T lymphocytes in the subcutaneous tumor samples of mice. **E** The effect of the expression levels of COL1A1, LAMA1, COL4A1, and USP8 on biochemical recurrence-free survival. **F** Correlation analysis of the IHC relative expression levels of NBR1, MHC-1, and PD-L1 with USP8. **G** Correlation analysis of the IHC relative expression levels of COL1A1, LAMA1, and COL4A1 with USP8. **H**, **I** Correlation analysis of the IHC relative expression levels of COL1A1, LAMA1, COL4A1 with PD-L1 and MHC-1. **J** The model showing the extracellular matrix-derived mechanical stiffness promotes the progression and immune evasion of PCa via USP8-mediated MHC-1 degradation and PD-L1 abundance. Data in C were analyzed by two-way repeated measures ANOVA test. Data are presented as the mean ± SD of at least three independent experiments and were analyzed with one-way ANOVA test unless otherwise stated, *P < 0.05, **P < 0.01, ***P < 0.001.

Next, we investigated whether USP8 can be used as a target for sensitizing immunotherapy of PCa. RM-1 cells were injected into the flanks of C57BL/6J mice and randomly divided into four group: the untreated control group, the USP8 inhibitor group, the anti-PD-L1 therapy group, the USP8 inhibitor and anti-PD-L1 combination therapy group. The results demonstrated that the USP8 inhibitor and anti-PD-L1 combination therapy could significantly inhibit the tumor growth rate (Fig. 9A–C) and increase the proportion of CD3^+^ T lymphocytes in CD45^+^ cells, CD8^+^ T lymphocytes in CD45^+^CD3^+^ cells, and the proportion of GZMB^+^ cells or PRF1^+^ cells in CD8^+^ T lymphocytes in the subcutaneous tumor, suggesting the potential application value of USP8 inhibitor in sensitizing immunotherapy of PCa (Fig. 9D). The survival analysis suggested that patients with high expression of COL1A1, LAMA1, COL4A1, USP8 has lower biochemical recurrence-free survival compared with those with low expression (Fig. 9E). The IHC relative expression of USP8 shows positive correlation with NBR1, PD-L1, but negative correlation with MHC-1 (Fig. 9F), and the correlation analysis showed that the IHC relative expression of COL1A1, LAMA1, and COL4A1 shows positive correlation with USP8 (Fig. 9G). Besides, the correlation analysis also showed that the IHC relative expression of COL1A1, LAMA1, and COL4A1 shows negative correlations with MHC-1 (Fig. 9H), and positive correlation with PD-L1 (Fig. 9I). The Fig. 9J presented that the model showing the extracellular matrix-derived mechanical stiffness promotes the progression and immune evasion of PCa via USP8-mediated MHC-1 degradation and PD-L1 abundance.

## Discussion

Gleason score has been acknowledgedly and widely used in clinical practice to evaluate the invasiveness and aggressiveness of PCa, and higher Gleason score represents higher invasiveness and aggressiveness of PCa. [12]. The mechanical stiffness and structural/bioactive proteins of ECM have been proved to be critical to the cell characteristics [30]. Besides, it has been reported that the changes of physical stiffness in the tumor microenvironment showed vital role in regulating malignant biological properties of various solid tumors, such as, gene expression, cell morphology, migration, and malignancy [31–33]. It has also been reported that higher Gleason score represents higher ECM mechanical stiffness in PCa [11]. In this study, the different levels of extracellular ECM stiffness were also observed in PCa tissues with different Gleason score. Besides, we found that the deposition of matrix associated proteins might be one of the vital causes of stiffness increase in PCa tissue with the increase of Gleason score. More importantly, we revealed that high PAAG stiffness plays a vital role in changing cellular morphology, inhibiting apoptosis, accelerating the proliferation, migration, invasion, EMT, and immune evasion of PCa cells. These results were consistent with other tumors [34], and increased our interest to explore the specific molecular mechanism of ECM stiffness enhancing the progression and immune evasion of PCa.

Integrin β1/FAK/YAP axis is a signaling pathway of mechanical sensor and regulator and has been confirmed to modulate the biological behavior of tumor cells and stromal cells [35, 36]. In this study, we also found that the integrin β1/FAK/YAP axis in PCa was significantly activated with the increase of PAAG stiffness and Gleason score. The YAP protein is also a recognized transcriptional coactivator in response to mechanical signal, and p-YAP is an inactivated form localized in cytoplasm while YAP is an activated form localized in cell nucleus to act as a transcriptional coactivator [20]. We found that the YAP protein could translocate into cell nucleus and bind to TEAD2 to act as a transcription coactivator in response to higher PAAG stiffness in PCa. These results suggested the vital role of integrin β1/FAK/YAP axis in PCa in response to PAAG stiffness. Next, the results of RNA-seq identified USP8 as the downstream target gene regulated by the PAAG stiffness and high PAAG stiffness upregulated USP8 via integrin β1/FAK/YAP axis. In vivo and in vitro experiment demonstrated that the upregulation of USP8 could promote the progression and immune evasion of PCa, with is consistent with Islam’ study [37]. Besides, high PAAG stiffness promoted the progression and immune evasion of PCa via USP8. The role of USP8 in the cancer progression has been reported in several types of cancers. Li, et al. uncovered that USP8 counteracted ferroptosis by stabilizing GPX4 and USP8 might be a promising therapeutic target for enhancing cancer immunotherapy [38]. Tang, et al. found that USP8 positively promoted the tumorigenesis of hepatocellular carcinoma through β-catenin stabilization [39]. Xie, et al. revealed that USP8 promoted the progression of cancer and CD8 + T cell exhaustion by deubiquitinating the TGF-β receptor TβRII [40]. These reports were consistent with our study.

Deubiquitinating enzymes (DUBs) can remove ubiquitin chains from substrates, thereby stabilizing target proteins and affecting biological processes [41]. In the human genome, there were approximately 100 DUBs and USPs is the well-defined largest subfamily with nearly 60 members [42]. Recently, the vital roles of the stabilization of oncoproteins promote numerous studies related to the functions of USPs in cancer progression and immune evasion [43, 44]. Wang et al. demonstrated that USP22 directly regulated PD-L1 stability through de-ubiquitination and played an important role in PD-L1 mediated immune evasion [45]. Xiao et al. revealed that USP5 governed PD-1 homeostasis via de-ubiquitination and could serve as a potential combinatorial therapeutic target for promoting anti-tumor immunotherapy [44]. Xie et al. found that USP8 could promote the progression of cancers and the exhaustion of CD8 + T cell by deubiquitinating TβRII and USP8 inhibitors could simultaneously suppress the cancer progression and improve the efficacy of cancer immunotherapy [40]. In our study, to investigate the downstream molecular mechanism of USP8, we performed 4D label free quantitative proteomics and ubiquitinome and identified NBR1 as a specifically targeted de-ubiquitinating substrate of USP8. Further researches revealed that USP8 could specifically interact with NBR1 protein, and affect the stability of endogenous NBR1 protein and upregulate the expression level of NBR1 protein via K63-linked de-ubiquitination. USP8 promoted the PCa progression and immune evasion through upregulating NBR1.

MHC-1 molecule has been reported to degrade through NBR1-mediated selective autophagy to enhance the tumor cells escaped from CTLs in glioma and pancreatic cancer [24, 25]. Here, we found that MHC-1 was downregulated with the increase of Gleason score and PAAG stiffness of PCa. Besides, MHC-1 was also found to bind to NBR1 and degrade via autophagy-lysosome pathway in PCa cells. The expression level and stability of MHC-1 could be regulated by the PAAG stiffness via USP8/NBR1 axis. These results indicated that the PAAG stiffness could promote the progression and immune evasion via USP8/NBR1-mediated degradation of MHC-1. The increased abundance of PD-L1 could weaken the immune response of immune system, and PD-L1 has been reported as a downstream deubiquitinating substrate of USP8 in pancreatic cancer [22] and non-small cell lung cancer [29]. In our study, we found that PD-L1 was upregulated with the increase of Gleason score and PAAG stiffness of PCa. Further research revealed that high PAAG stiffness could promote the stabilization of PD-L1 protein via USP8-mediated K48-de-ubiquitination in PCa. Take together, our study demonstrated that the degradation of MHC-1 and abundance of PD-L1 in PCa were responsible for the direct causes of PCa immune evasion, and high PAAG stiffness-induced USP8 high expression is the main molecular mechanism of the degradation of MHC-1 and abundance of PD-L1 in PCa. In addition, the USP8 inhibitor and anti-PDL1 combination therapy could significantly inhibit the tumor growth rate, suggesting the potential application value of USP8 inhibitor in sensitizing immunotherapy of PCa.

However, there were several limitations of this study. The major limitation is that the in vitro PAAG model used in this study to simulate the ECM stiffness is a 2D plane model, which could not reflect the complex three-dimensional tumor microenvironment of PCa. Secondly, we do not accurately measure the stiffness of the PAAG model by atomic force microscopy. Finally, the clinical application of USP8 inhibitor need further verification.

## Conclusion

High ECM stiffness promotes the progression and immune evasion of PCa via integrin β1/FAK/YAP axis-mediated USP8 upregulation. Increased expression of USP8 plays a vital role in regulating abnormal signal of MHC-1/TCR complex and PD-L1/PD-1 complex via its de-ubiquitination. The USP8 inhibitor presents a potential application value in sensitizing immunotherapy of PCa.

## Supplementary information

Supplementary materials information

Table S1

Table S2

Figure S1

Figure S2

Figure S3

Figure S4

Figure S5

Figure S6

Figure S7

Figure S8

Figure S9

Original data of WB

## Data Availability

The data that support the findings of this study are available from the corresponding author upon reasonable request.
