# Supplementary material for: Mechanical signal modulates prostate cancer immune escape by USP8-mediated ubiquitination-dependent degradation of PD-L1 and MHC-1
Source: Cell Death Dis. 2025 May 23;16(1):413. doi: 10.1038/s41419-025-07736-4 (PMC12102395; doi:10.1038/s41419-025-07736-4)
Supplement: Supplementary file 1 — Supplementary materials information [file 41419_2025_7736_MOESM1_ESM.docx]

**Supplementary materials**

**Figure S1. Strain-stress curves, elastic modulus calculations, and macroscopic observations of PAAG from different angles and thin slices (0.7mm)** **developed by texture analyzer.**

**(A-C)** Low stiffness of polyacrylamide hydrogel (PAAG) model, **(D-F)** medium stiffness of PAAG model, **(G-I)** high stiffness of PAAG model. The values of low, medium, and high PAAG stiffness were defined as 2-3 kPa, 15-17 kPa, and 53-57 kPa, respectively.

**Figure S2. The effect of PAAG stiffness on the morphology and biological behavior of PCa cells. (A)** The microscopically morphological changes of DU145 and PC-3 cells cultured in different PAAG stiffness. **(B)** The phalloidine assay indicating changes of cytoskeleton morphology of DU145 and PC-3 cells cultured in different PAAG stiffness. **(C)** CCK8 assay showing the cell viability of DU145 and PC-3 cells cultured in different PAAG stiffness. **(D)** Representative images and quantitative results of EdU assay of DU145 and PC-3 cells cultured in different PAAG stiffness. **(E)** Representative images and quantitative results of colony formation assay showing the clone formation capability of DU145 and PC-3 cells cultured in different PAAG stiffness. **(F)** Western blot indicating the relative expression of PCNA and Cyclin D1 protein in DU145 and PC-3 cells cultured in different PAAG stiffness. **(G)** Flow cytometry analysis detecting the proportion of apoptosis of DU145 and PC-3 cells cultured in different PAAG stiffness. **(H)** Western blot indicating the relative expression of N-cadherin, E-cadherin and vimentin protein in DU145 and PC-3 cells cultured in different PAAG stiffness. **(I-J)** Representative images and quantitative results of Transwell migration and invasion assay of DU145 and PC-3 cells cultured in different PAAG stiffness. Data in C were analyzed by two-way repeated measures ANOVA test. Data are presented as the mean ± SD of at least three independent experiments and were analyzed with one-way ANOVA test unless otherwise stated, **P*<0.05, ***P*<0.01, ****P*<0.001.

**Figure S3. The screening of USP8 and its transcription factor in response to PAAG stiffness.**

Volcano plots present the differentially expressed genes (DEGs) by comparing the RNA-seq results **(A)** between low and medium stiffness, **(B)** between medium and high stiffness, **(C)** between low and high stiffness. **(D)** RT-qPCR and **(E)** western blotting indicating the efficiency of knockdown or overexpression of TEAD1, TEAD2, TEAD3, TEAD4 in DU145 and PC-3 cells cultured in plastic wells. **(F)** RT-qPCR indicating the effect of TEAD1, TEAD2, TEAD3, TEAD4 knockdown or overexpression on USP8 mRNA expression in DU145 and PC-3 cells cultured in plastic wells. Data are presented as the mean ± SD of at least three independent experiments and were analyzed with one-way ANOVA test unless otherwise stated, **P*<0.05, ***P*<0.01, ****P*<0.001.

**Figure S4. USP8 promotes the proliferation, migration, invasion and immune evasion of PCa cells.**

**(A-B)** RT-qPCR and western blot indicating the efficiency of USP8 knockdown or overexpression in DU145 and PC-3 cells. **(C)** CCK8 assay indicating the effect of USP8 knockdown or overexpression on the cell viability of DU145 and PC-3 cells. **(D)** The colony formation assay showing the effect of USP8 knockdown or overexpression on the clone formation capability of DU145 and PC-3 cells. **(E-F)** Representative images and quantitative results of Transwell migration and invasion assay indicating the effect of USP8 knockdown or overexpression on the migration and invasion ability of DU145 and PC-3 cells. **(G-H)** The quantitative results of the cytotoxic T cell killing assay indicating the effect of USP8 knockdown or overexpression on the cytotoxic T cell killing ability of CD8+T cells to DU145 and PC-3 cells. **(I-K)** RM-1 stable cells of indicated groups were injected into the flank of mice. Tumor volumes were measured every 3 days. Tumor images, weight and growth curves were obtained at day 18 after dissection. **(L)** Flow cytometry was used to quantitatively analyze the proportion of CD3^+^ T lymphocytes in CD45^+^ cells, CD8^+^ T lymphocytes in CD45^+^CD3^+^ cells, and the proportion of GZMB^+^ cells or PRF1^+^ cells in CD8^+^ T lymphocytes in the subcutaneous tumor samples of mice. The X axis represents the different group. Data in C and K were analyzed by two-way repeated measures ANOVA test. Data are presented as the mean ± SD of at least three independent experiments and were analyzed with unpaired two-tailed student’s t-test or one-way ANOVA test unless otherwise stated, **P*<0.05, ***P*<0.01, ****P*<0.001.

**Figure S5. NBR1 was ubiquitinated by** **TRIM21 and then degraded via interacting with P62/SQSTM1 through autophagy-lysosome pathway.**

**(A)** Endogenous ubiquitination assay of NBR1 in DU145 and PC-3 cells treated with 3-MA for 6 h. **(B)** Western blot analysis of NBR1 expression in DU145 and PC-3 cells treated with 3-MA or MG132. **(C-D)** Mass spectrometry after immunoprecipitation identified a total of five E3 ubiquitin ligase of NBR1 but only TRIM21 was validated by Co-immunoprecipitation. **(E)** Endogenous ubiquitination assay of NBR1 in TRIM21 knockdown DU145 and PC-3 cells treated with 3-MA for 6 h. **(F-G)** Mass spectrometry after immunoprecipitation identified a total of two Autophagy cargo receptors of NBR1 but only P62 was validated by Co-immunoprecipitation. Data are presented as the mean ± SD of at least three independent experiments and were analyzed with unpaired two-tailed student’s t-test or one-way ANOVA test unless otherwise stated, **P*<0.05, ***P*<0.01, ****P*<0.001.

**Figure S6. Validation of K63 ubiquitination of NBR1 in PCa cells.**

(A) Ubiquitination assay of NBR1 in subcutaneous tumors derived from RM-1 cells in different PAAG stiffness or in combination with shUSP8 transduction. (B) Endogenous ubiquitination assay of NBR1 using K63-linked or K48-linked specific polyubiquitin antibody in DU145 and PC-3 cells treated with 3-MA for 6 h.

**Figure S7. USP8 promotes the proliferation, migration, invasion and immune evasion of PCa cells via upregulating NBR1.**

**(A)** CCK8 assay showing the cell viability of DU145 and PC-3 cells transduced with USP8 shRNA or in combination with Myc-NBR1 plasmid transduction. **(B)** Representative images and quantitative results of colony formation assay of DU145 and PC-3 cells transduced with USP8 shRNA or in combination with Myc-NBR1 plasmid transduction. **(C-D)** Transwell migration and invasion assay of DU145 and PC-3 cells transduced with USP8 shRNA or in combination with Myc-NBR1 transduction. **(E-F)** The quantitative results of the cytotoxic T cell killing assay indicating the effect of USP8 shRNA or in combination with Myc-NBR1 transduction on the cytotoxic T cell killing ability of CD8+T cells to DU145 and PC-3 cells. Data in A were analyzed by two-way repeated measures ANOVA test. Data are presented as the mean ± SD of at least three independent experiments and were analyzed with unpaired two-tailed student’s t-test or one-way ANOVA test unless otherwise stated, **P*<0.05, ***P*<0.01, ****P*<0.001.

**Figure S8. PAAG stiffness downregulated the expression of MHC-1 via USP8/NBR1 axis.** Western blot indicating the relative expression of MHC-1 protein in DU145 and PC-3 cells cultured in different PAAG stiffness and in combination with 3-MA treatment or shUSP8 transduction or shNBR1 transduction or shNBR1 plus Flag-USP8 transfection or shNBR1 plus rapamycin treatment or Myc-NBR1 transfection or Myc-NBR1-K887R transfection. Data are presented as the mean ± SD of at least three independent experiments and were analyzed with unpaired two-tailed student’s t-test or one-way ANOVA test unless otherwise stated, *****P*<0.001.

**Figure S9. Validation of K48 ubiquitination of PD-L1 in PCa cells.**

(A) Ubiquitination assay of PD-L1 in subcutaneous tumors derived from RM-1 cells in different PAAG stiffness or in combination with shUSP8 transduction. (B) Endogenous ubiquitination assay of PD-L1 using K63-linked or K48-linked specific polyubiquitin antibody in DU145 and PC-3 cells treated with MG132 for 6 h.

**Table S1. Sequences of primers used for RT-qPCR.**

**Table S2. siRNA and shRNA sequences.**
