# Supplementary material for: Mechanical signal modulates prostate cancer immune escape by USP8-mediated ubiquitination-dependent degradation of PD-L1 and MHC-1
Source: Cell Death Dis. 2025 May 23;16(1):413. doi: 10.1038/s41419-025-07736-4 (PMC12102395; doi:10.1038/s41419-025-07736-4)
Supplement: Supplementary file 2 — Table S1 [file 41419_2025_7736_MOESM2_ESM.docx]

| **Table S1. Sequences of primers used for RT-qPCR.** | |
| --- | --- |
| **Genes** | **Sequences** |
| **TEAD1** |  |
| F | 5’- GCAAGGTTTGAGAATGGCCG -3’ |
| R | 5’- CACACAGGCCATGCAGAGTA -3’ |
| **TEAD2** |  |
| F | 5’- CACCGTTCACCTTGTCACTG -3’ |
| R | 5’- GTGCCTCTGGTAAGAATCAACTG -3’ |
| **TEAD3** |  |
| F | 5’- CCTGGAGGCAGTAGATGTGC -3’ |
| R | 5’- CTGGATGGTGCTGTTGAGGT -3’ |
| **TEAD4** |  |
| F | 5’- TCCACAAGCTCAAGCACCTC -3’ |
| R | 5’- CAGCAAGGTCTCCTGTGTGT -3’ |
| **USP8** |  |
| F | 5’- AAGGTGAAGTGGCAGAAGAATT -3’ |
| R | 5’- TGTGAATCTTGCTGACTGTATCC -3’ |
| **NBR1** |  |
| F | 5’- ACGTAAAGCAGAGGTCAAGGA -3’ |
| R | 5’- CACTGAGCATTGGCATAACGG -3’ |
| **PD-L1** |  |
| F | 5’- AGGCCGAAGTCATCTGGACA -3’ |
| R | 5’- TGTTGATTCTCAGTGTGCTGGT -3’ |
| **YAP** |  |
| F | 5’- CCTGAACAGTGTGGATGAGATG -3’ |
| R | 5’- AGGAATGGCTTCAAGGTAGTCT -3’ |
