# Supplementary material for: Mechanical signal modulates prostate cancer immune escape by USP8-mediated ubiquitination-dependent degradation of PD-L1 and MHC-1
Source: Cell Death Dis. 2025 May 23;16(1):413. doi: 10.1038/s41419-025-07736-4 (PMC12102395; doi:10.1038/s41419-025-07736-4)
Supplement: Supplementary file 3 — Table S2 [file 41419_2025_7736_MOESM3_ESM.docx]

| **Table S2. siRNA and shRNA sequences** | | |
| --- | --- | --- |
| **Product** | **Sense (5’-3’)** | **Antisense (5’-3’)** |
| siYAP #1 | GUCAGAGAUACUUCUUAAATT | UUUAAGAAGUAUCUCUGACTT |
| siYAP #2 | CCACCAAGCUAGAUAAAGATT | UCUUUAUCUAGCUUGGUGGTT |
| siYAP #3 | CCAGAUGACUUCCUGAACATT | UGUUCAGGAAGUCAUCUGGTT |
| siITGB1 #1 | GGGCAUCAUUCAGAAGAUATT | UAUCUUCUGAAUGAUGCCCTT |
| siITGB1 #2 | GAACCUCGCAGUCAUUUAUTT | AUAAAUGACUGCGAGGUUCTT |
| siITGB1 #3 | CAGGUUUACUGAACUUAAATT | UUUAAGUUCAGUAAACCUGTT |
| siFAK #1 | GGCGUAACAAUAAGUUACATT | UGUAACUUAUUGUUACGCCTT |
| siFAK #2 | GGGUGGUGCACAAAUUCAATT | UUGAAUUUGUGCACCACCCTT |
| siFAK #3 | CCCUCCAGAUGACAUAGAATT | UUCUAUGUCAUCUGGAGGGTT |
| siTEAD1 #1 | GUUCUUGCCAGAAGGAAAUTT | AUUUCCUUCUGGCAAGAACTT |
| siTEAD1 #2 | GGCAUGCCAACCAUUCUUATT | UAAGAAUGGUUGGCAUGCCTT |
| siTEAD3 #1 | CUGGAGUAUUCAGCCUUCATT | UGAAGGCUGAAUACUCCAGTT |
| siTEAD3 #2 | GAGGAUUGAAGGAGCUCUATT | UAGAGCUCCUUCAAUCCUCTT |
| siTEAD4 #1 | CUCCCUGAGAAGUACAUGATT | UCAUGUACUUCUCAGGGAGTT |
| siTEAD4 #2 | GACAGAGUAUGCUCGCUAUTT | AUAGCGAGCAUACUCUGUCTT |
| shUSP8 #1 | CCACAGATTGATCGTACTAAA | / |
| shUSP8 #2 | GCTGTGTTACTAGCACTATAT | / |
| shUSP8 #3 | GCCAGAATGAAGAGGTGTCTA | / |
| shNBR1 #1 | GCCAGGAACCAAGTTTATCAA | / |
| shNBR1 #2 | CCATCCTACAATATCTGTGAA | / |
| shNBR1 #3 | GCAGTTAAACAGGGAAACCAA | / |
