# Supplementary figures and images for: Mechanical signal modulates prostate cancer immune escape by USP8-mediated ubiquitination-dependent degradation of PD-L1 and MHC-1
Source: Cell Death Dis. 2025 May 23;16(1):413. doi: 10.1038/s41419-025-07736-4 (PMC12102395; doi:10.1038/s41419-025-07736-4)

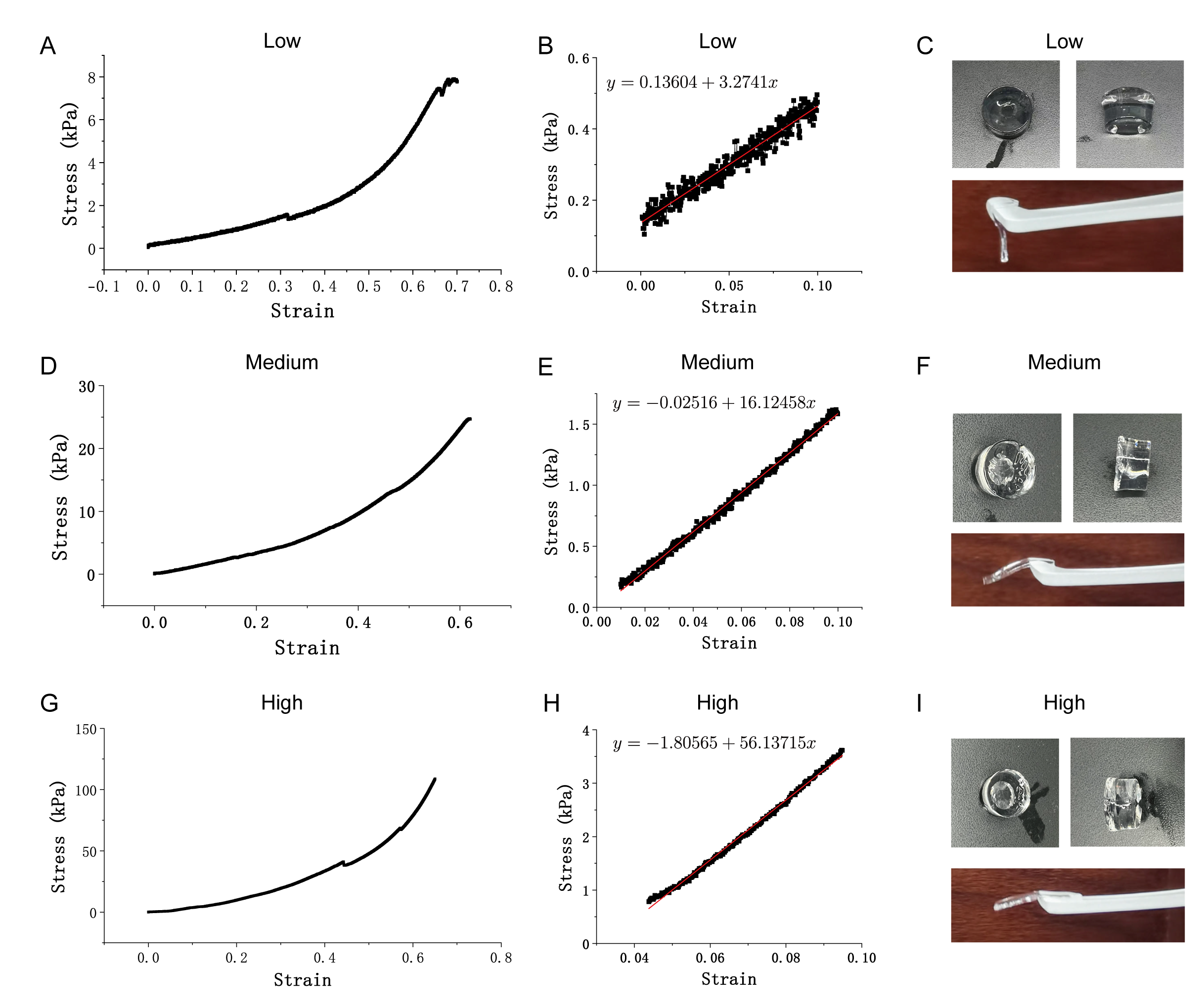

Supplement: Supplementary file 4 — Figure S1 [file 41419_2025_7736_MOESM4_ESM.tif]

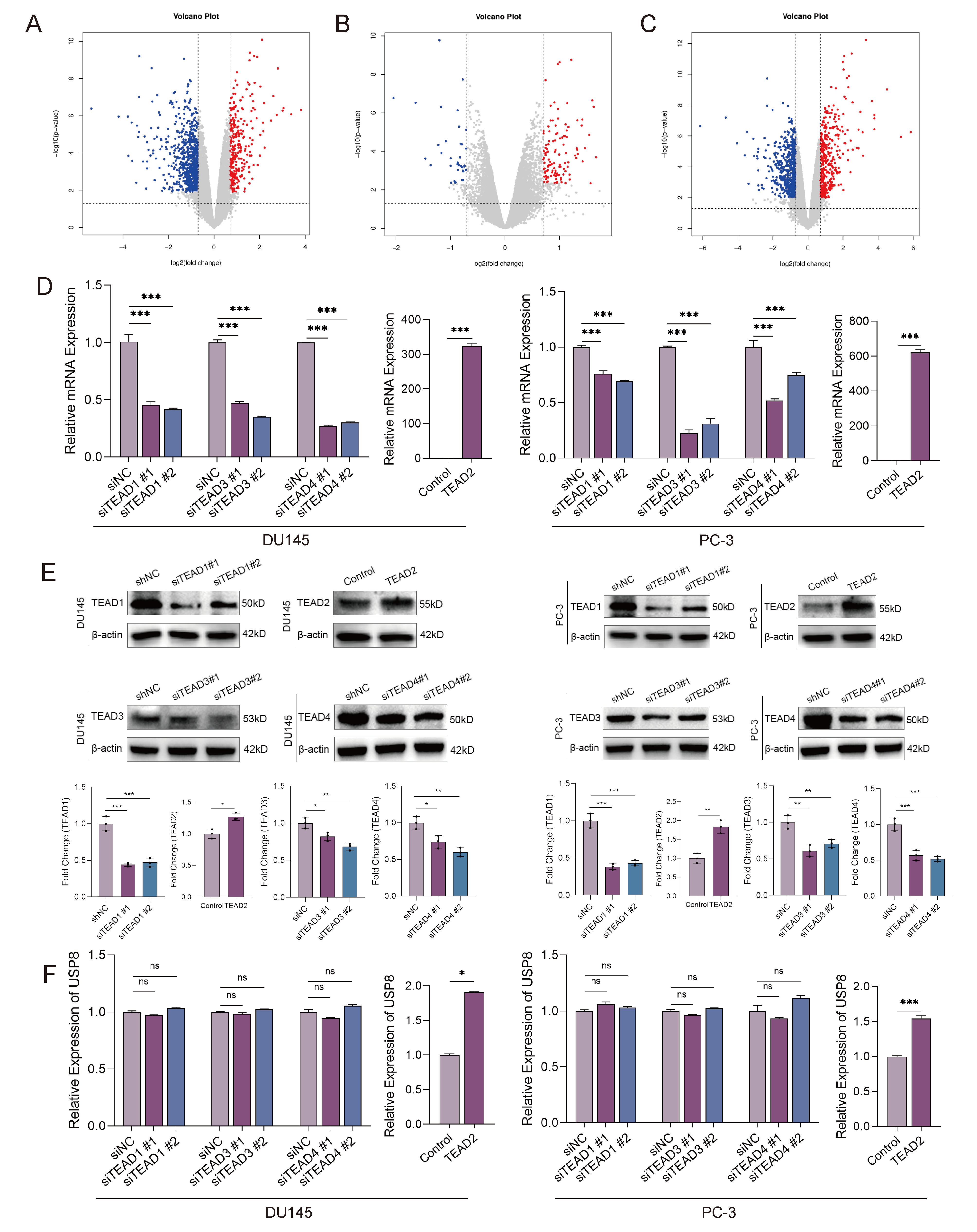

Supplement: Supplementary file 6 — Figure S3 [file 41419_2025_7736_MOESM6_ESM.tif]

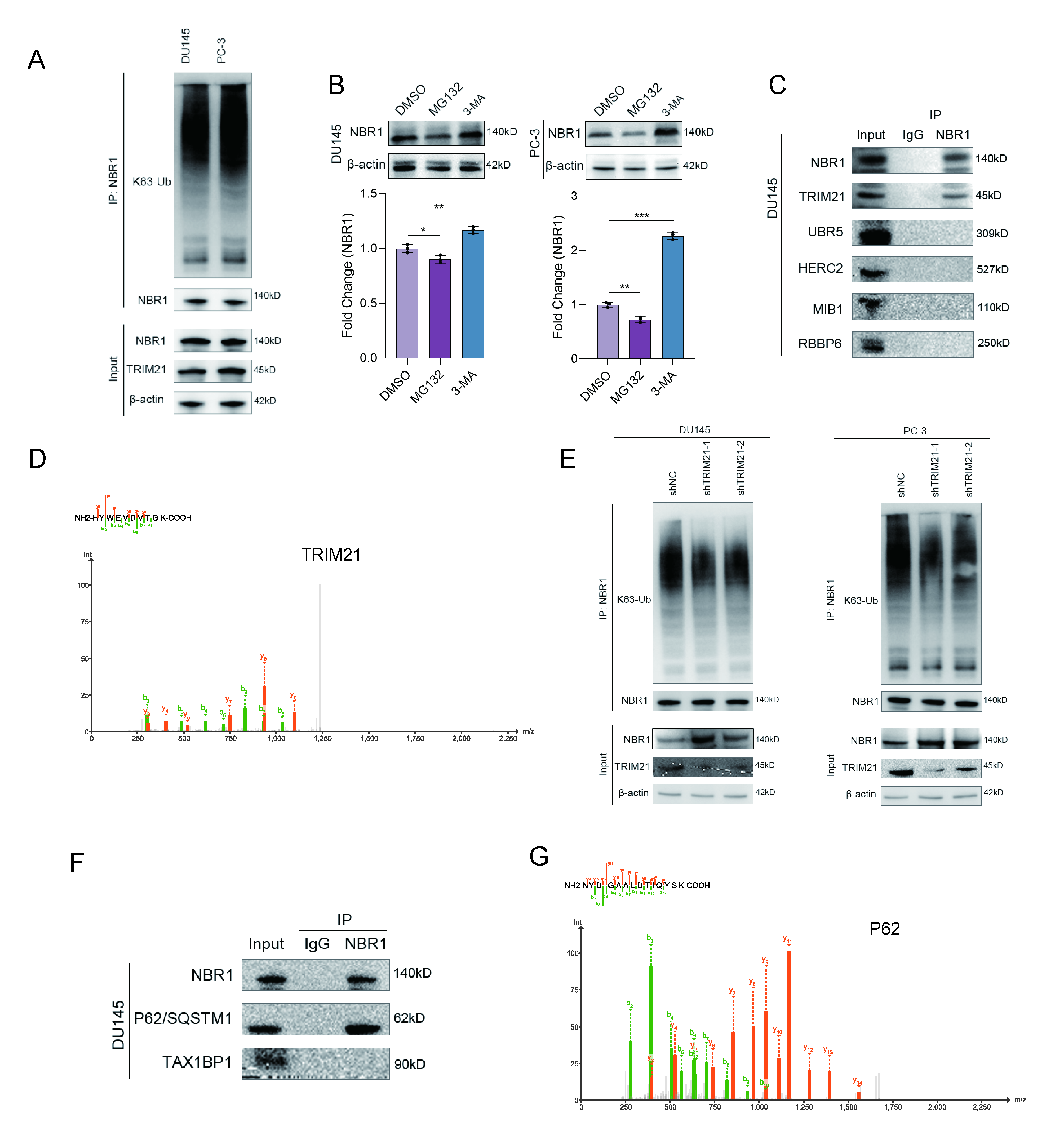

Supplement: Supplementary file 8 — Figure S5 [file 41419_2025_7736_MOESM8_ESM.tif]

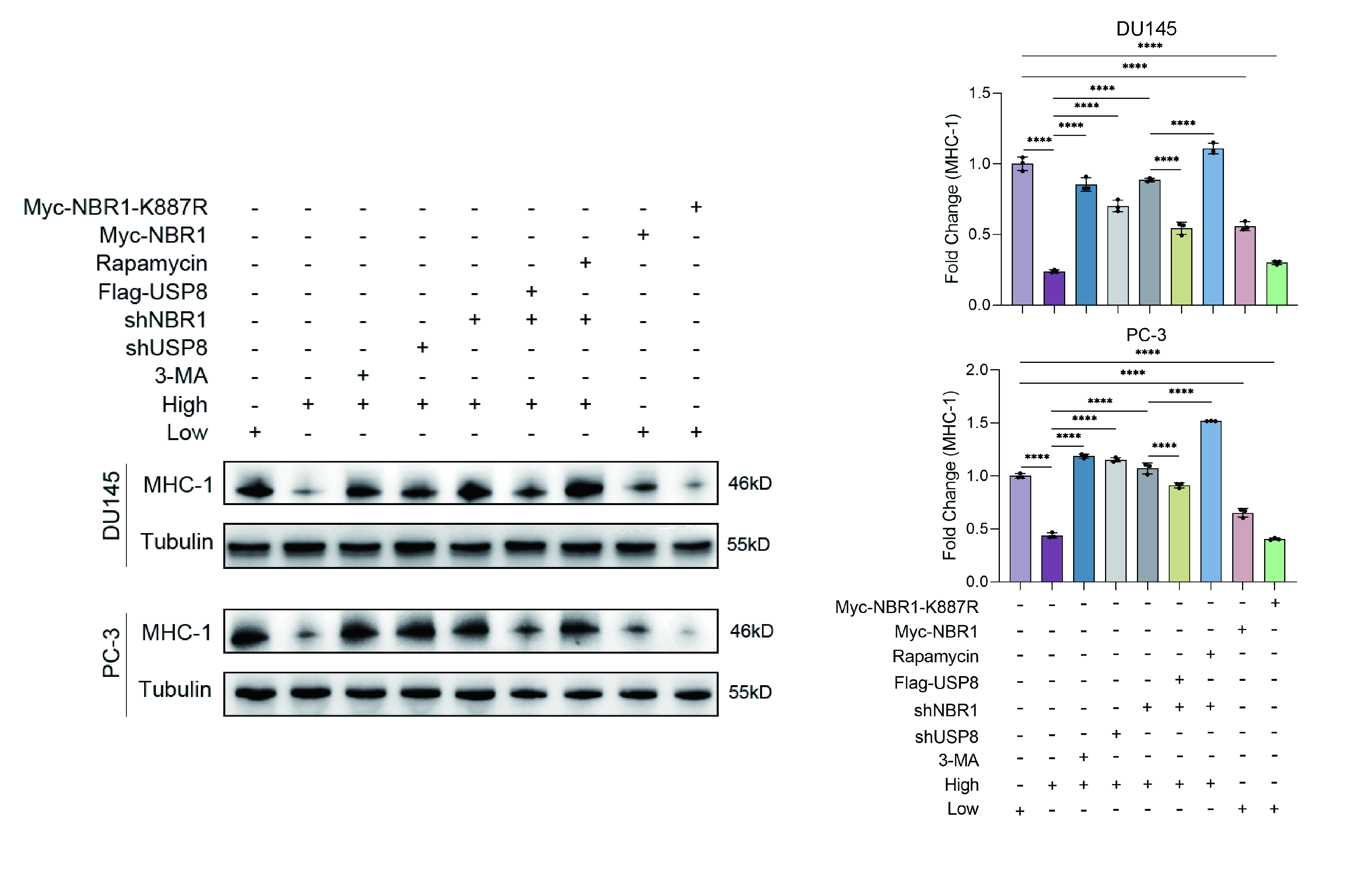

Supplement: Supplementary file 11 — Figure S8 [file 41419_2025_7736_MOESM11_ESM.tif]
