# Supplementary material for: Mechanical signal modulates prostate cancer immune escape by USP8-mediated ubiquitination-dependent degradation of PD-L1 and MHC-1
Source: Cell Death Dis. 2025 May 23;16(1):413. doi: 10.1038/s41419-025-07736-4 (PMC12102395; doi:10.1038/s41419-025-07736-4)

Figure 2A

Integrin β1

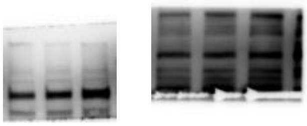

FAK

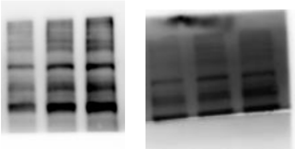

p-FAK

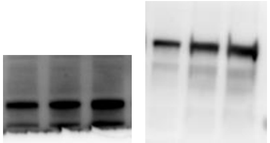

YAP

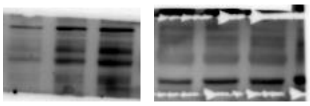

β-actin

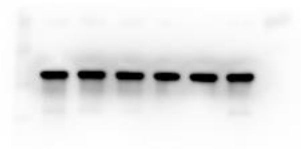

Figure 2D

pYAP

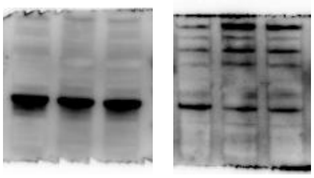

YAP

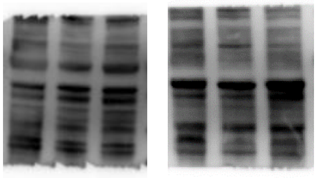

Figure 2G

DU145 USP8

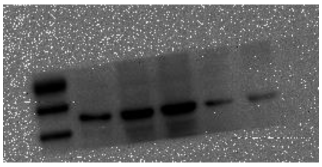

PC-3 USP8

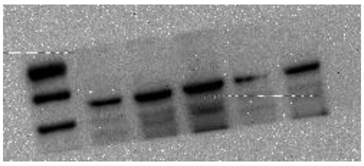

β-actin

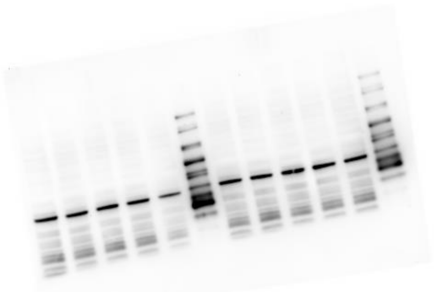

Figure 2I

DU145 USP8

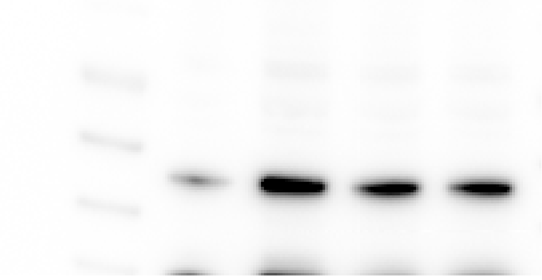

DU145 tubulin

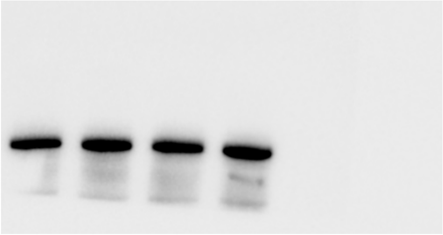

PC-3 USP8

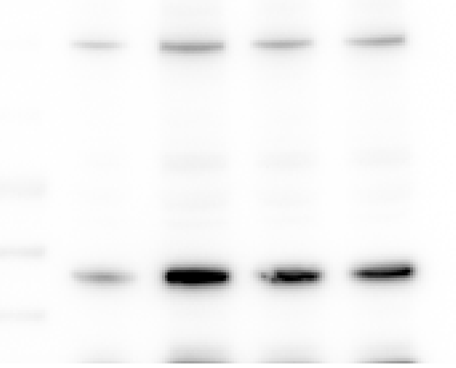

PC-3 tubulin

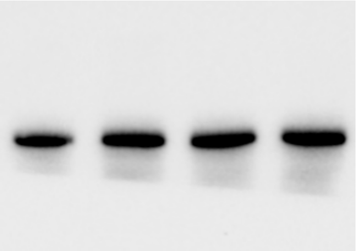

Figure 4J

Myc

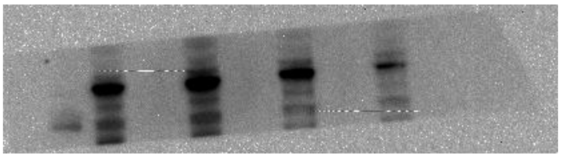

Flag

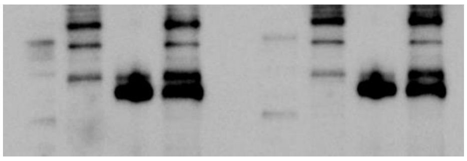

Figure 4K

IP_USP8_IB_USP8 (DU145 and PC-3)

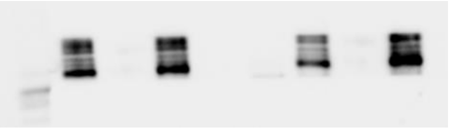

IP_USP8_IB_NBR1(DU145 and PC-3)

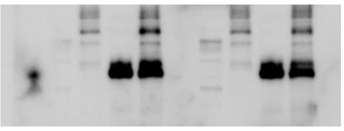

IP_NBR1_IB_ NBR1 (DU145 and PC-3)

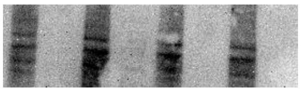

IP_ NBR1_IB_USP8 (DU145 and PC-3)

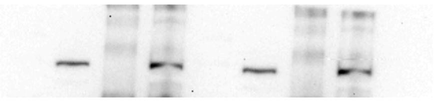

Figure 4N

USP8 (DU145 and PC-3)

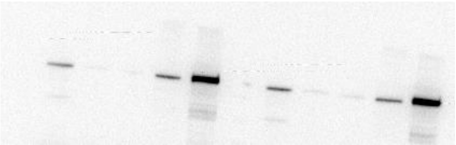

NBR1 (DU145 and PC-3)

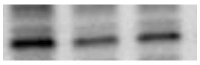

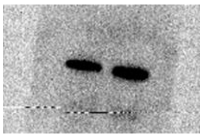

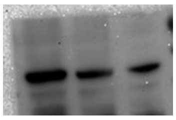

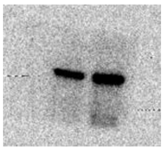

β-actin (DU145 and PC-3)

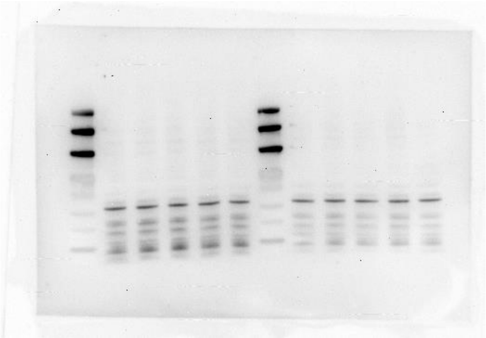

Figure 5A

IP_Myc-NBR1_IB_HA-Ub   IP_Myc-NBR1_IB_ Myc-NBR1

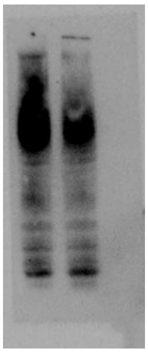

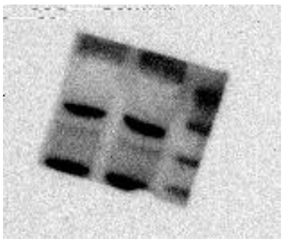

Input_ Myc-NBR1

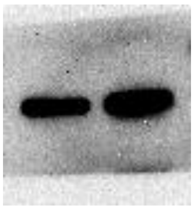

Input_Flag-USP8 Input_ Tubulin

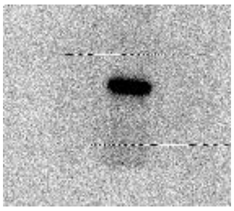

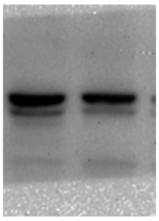

Figure 5B

IP_ NBR1_IB_Ub (DU145 and PC-3)

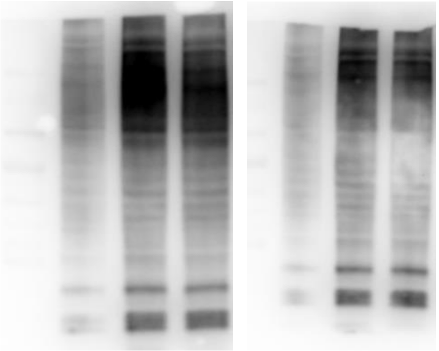

IP_ NBR1_IB_NBR1(DU145 and PC-3)

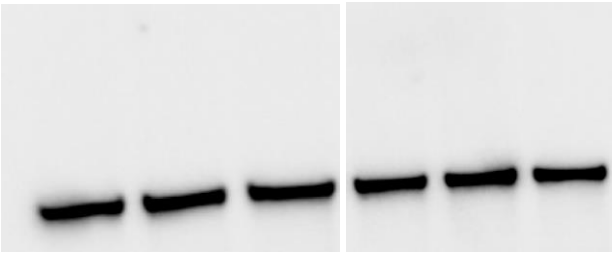

Input_ NBR1(DU145 and PC-3)

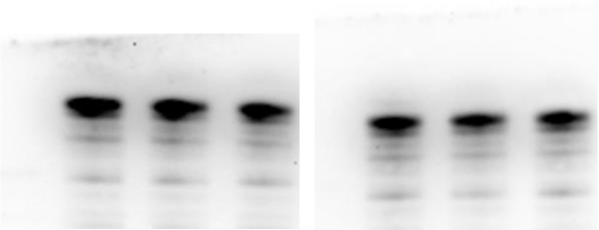

Input_USP8 (DU145 and PC-3)

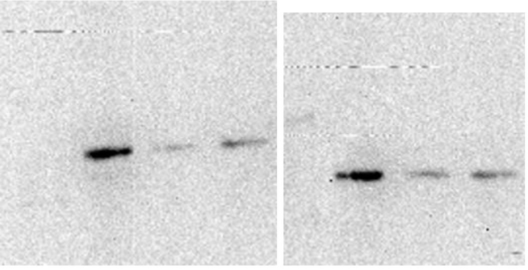

Input_ β-actin(DU145 and PC-3)

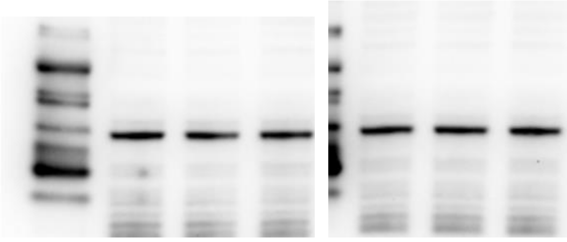

Figure 5C

HA-Ub Flag-USP8 Myc-NBR1

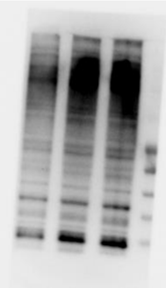

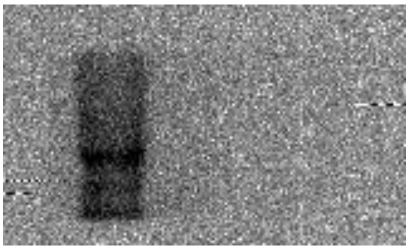

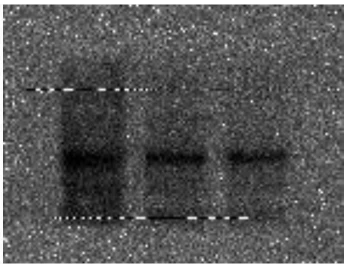

Figure 5D

IP_NBR1_IB_Ub

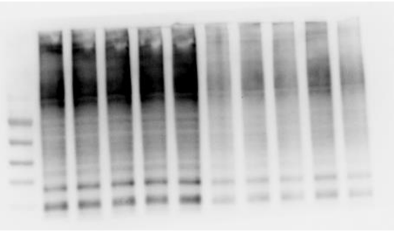

IP_ NBR1_IB_ NBR1

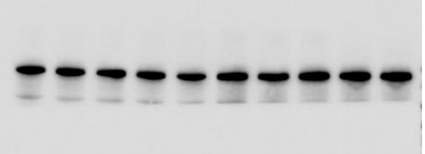

Input_ NBR1

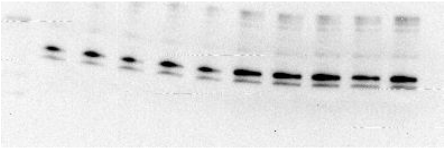

Input_USP8

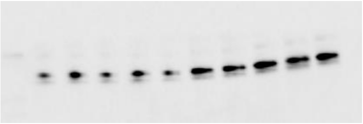

Input_β-actin

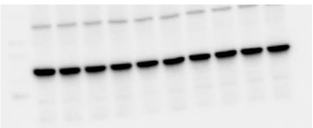

Figure 5E

IP_ NBR1_IB_Ub

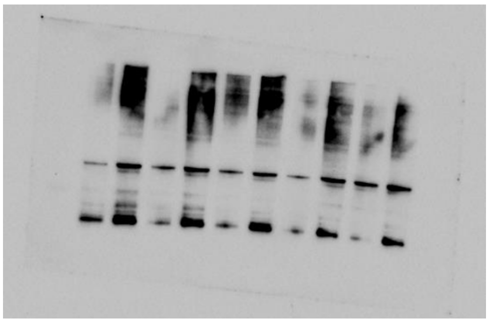

IP_ NBR1_IB_NBR1

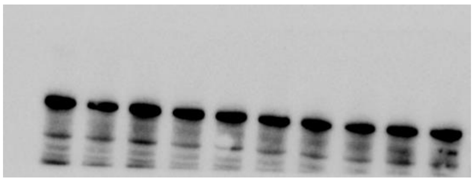

Input_ NBR1

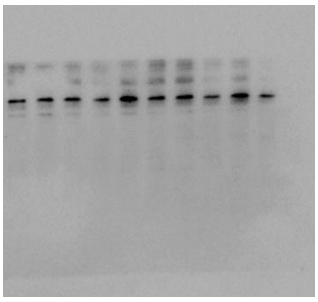

Input_USP8

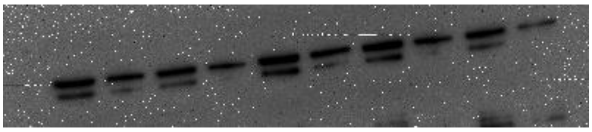

Input_Tubulin

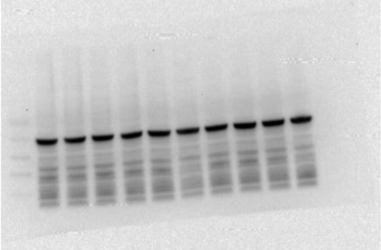

Figure 5F

IP_MYc-NBR1_IB_HA-Ub

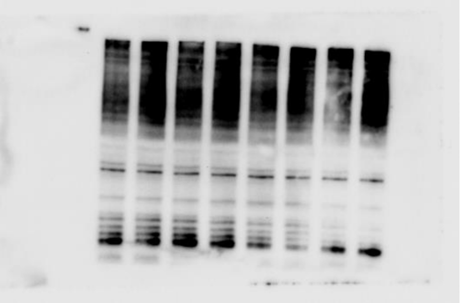

IP_ MYc-NBR1_IB_ MYc-NBR1

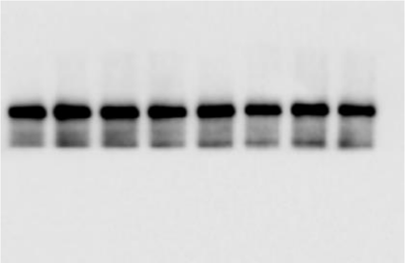

Input_ MYc-NBR1

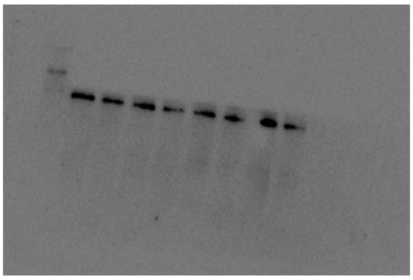

Input_Flag-USP8

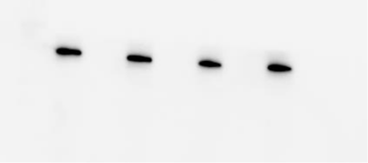

Input_β-actin

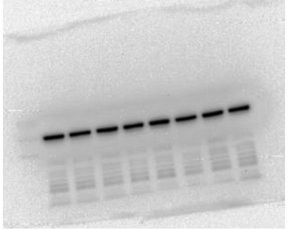

IP_MYc-NBR1_IB_HA-Ub

IP_ MYc-NBR1_IB_ MYc-NBR1

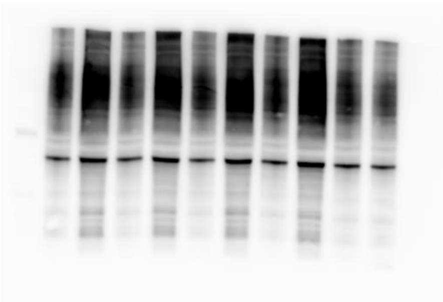

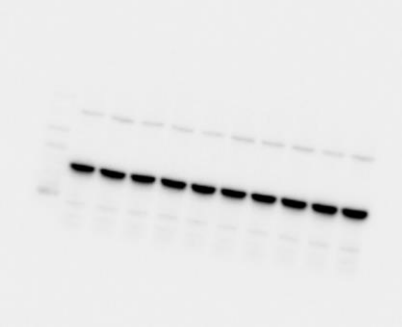

Input_ MYc-NBR1

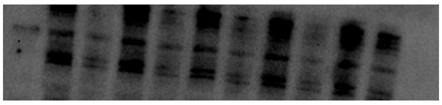

Input_Flag-USP8

Input_β-actin

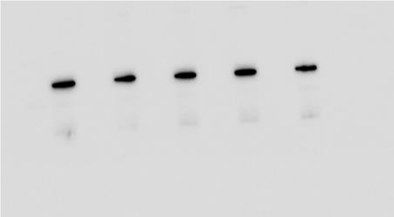

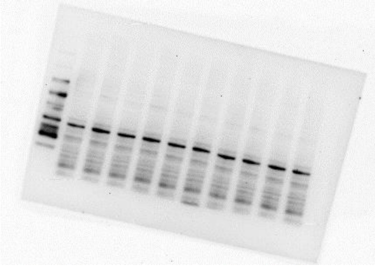

Figure 5G

IP_MYc-NBR1_IB_HA-Ub

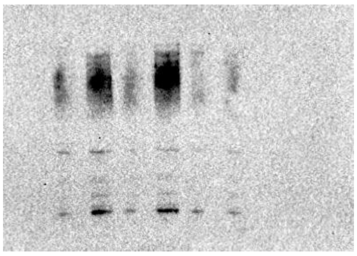

IP_ MYc-NBR1_IB_ MYc-NBR1

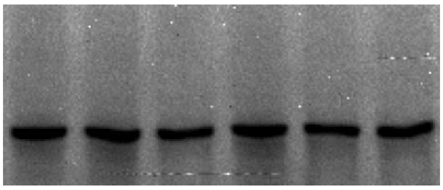

Input_ MYc-NBR1

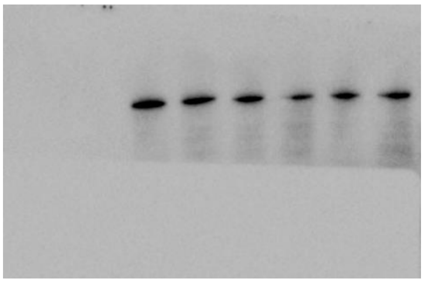

Input_Flag-USP8

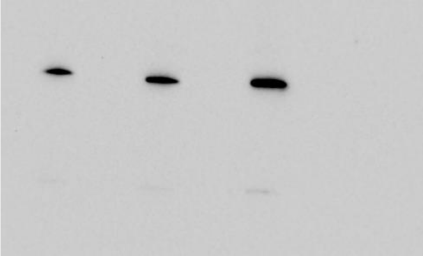

Input_β-actin

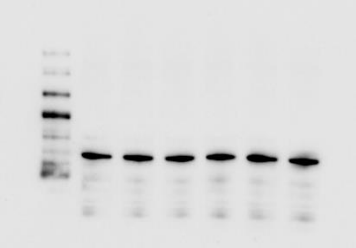

Figure 5H

USP8

NBR1

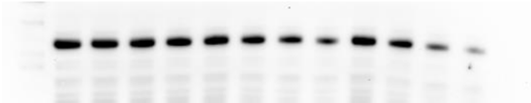

Tubulin

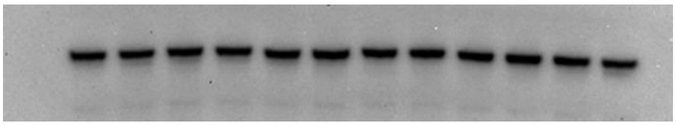

USP8

NBR1

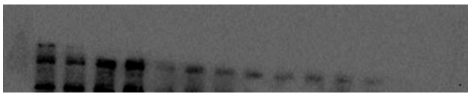

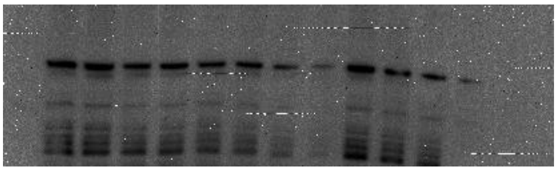

Tubulin

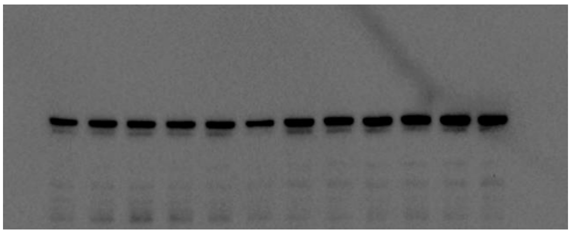

Figure 6A

IP_NBR1_IB_NBR1 (DU145 and PC3)   IP_NBR1_IB_MHC-1 (DU145 and PC3)

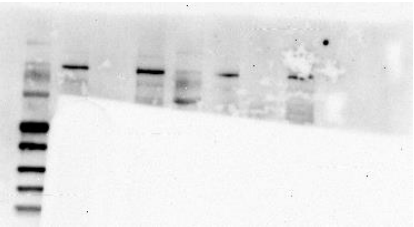

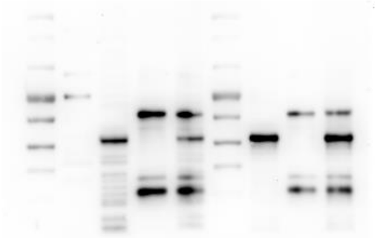

IP_MHC-1_IB_MHC-1 (DU145 and PC3)   IP_ MHC-1_IB_ NBR1 (DU145 and PC3)

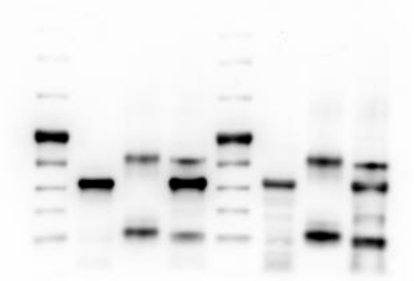

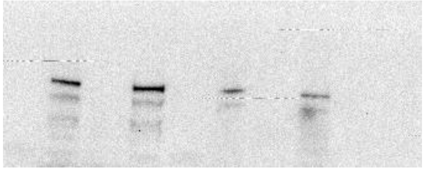

Figure 6B

MHC-1 (DU145 and PC3)   Tubulin (DU145 and PC3)

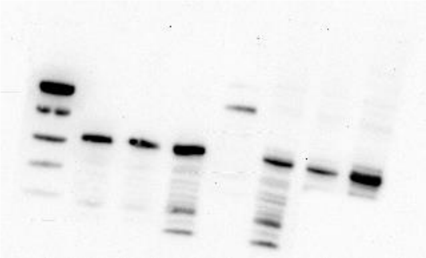

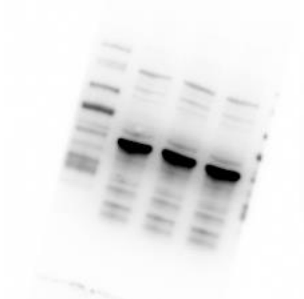

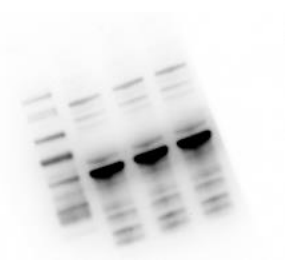

Figure 6C

IP_USP8_IB_USP8   IP_USP8_IB_MHC-1   IP_MHC-1_IB_ MHC-1   IP_MHC-1_IB_USP8

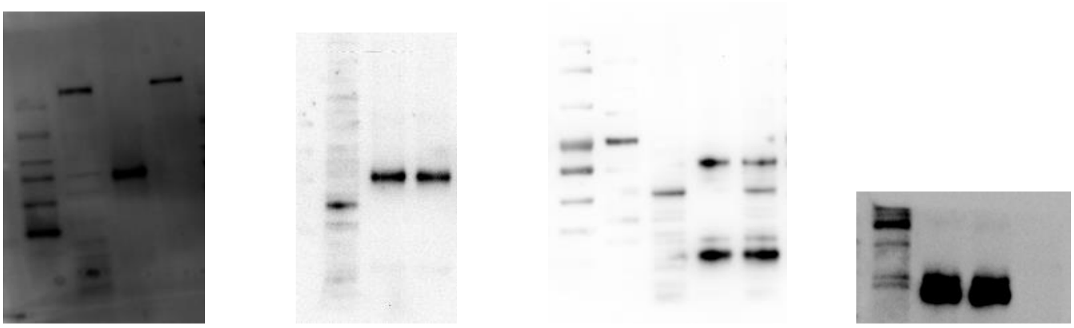

Figure 6D

USP8 (DU145 and PC3)

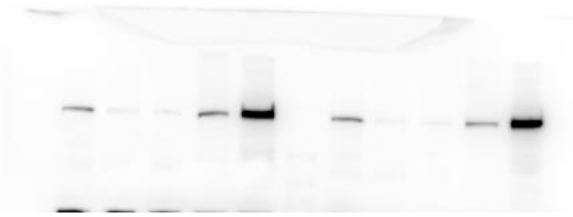

MHC-1 (DU145 and PC3)

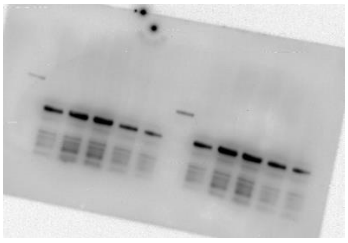

Tubulin (DU145 and PC3)

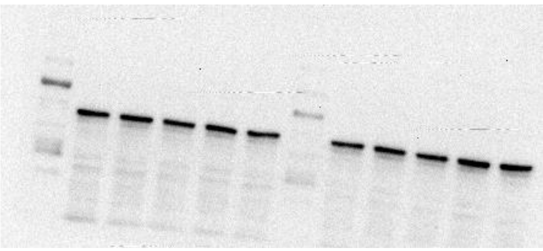

Figure 6E

MHC-1

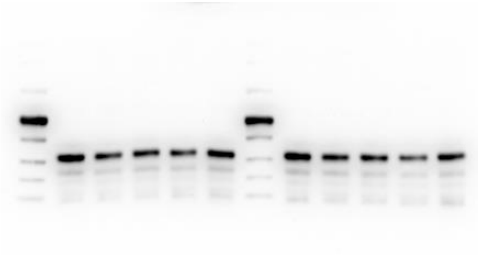

Tubulin

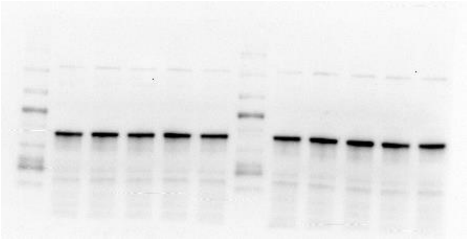

Figure 6F

MHC-1

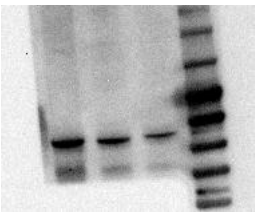

Tubulin

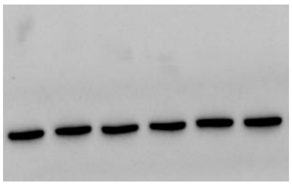

MHC-1

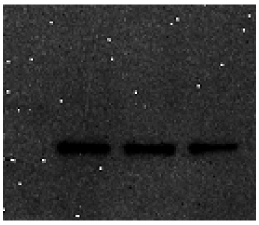

Figure 7A

His -PD-L1

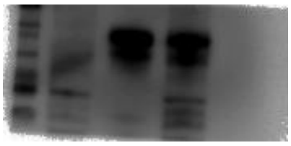

Flag-USP8

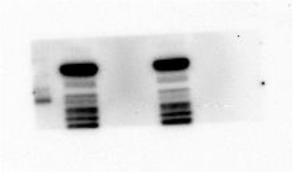

Flag-USP8

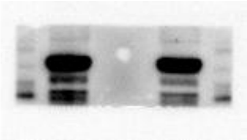

His-PD-L1

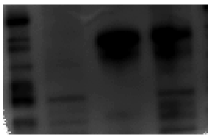

Figure 7B(DU145)

USP8

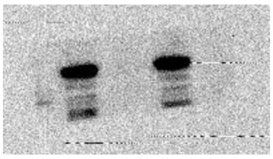

PD-L1

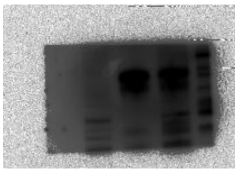

PD-L1

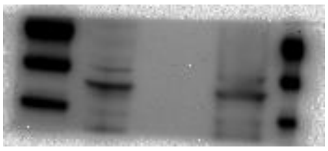

USP8

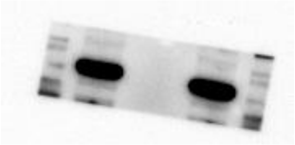

Figure 7B(PC-3)

USP8 **PD-L1**

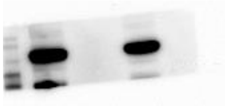

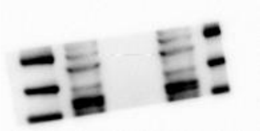

PD-L1

USP8

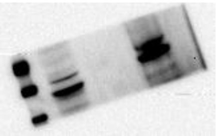

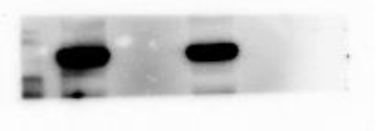

Figure 7D

PD-L1

Tubulin

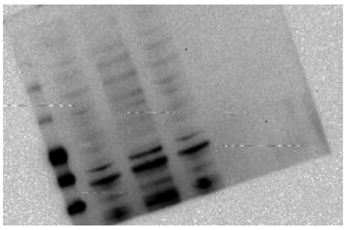

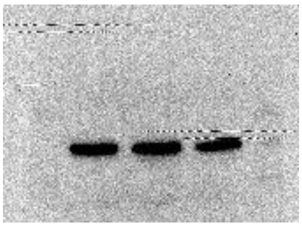

PD-L1

Tubulin

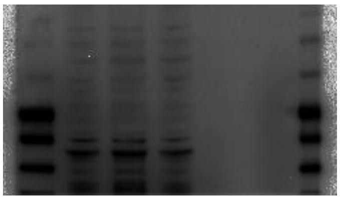

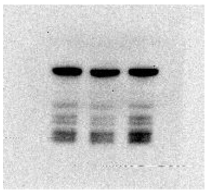

Figure 7F

USP8

PD-L1

Tubulin

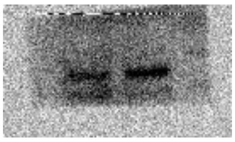

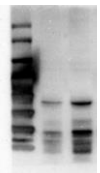

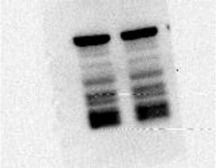

USP8

PD-L1

Tubulin

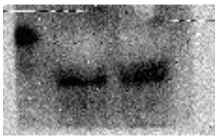

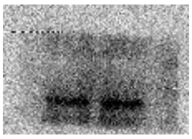

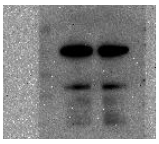

Figure 7F

USP8

PD-L1

Tubulin

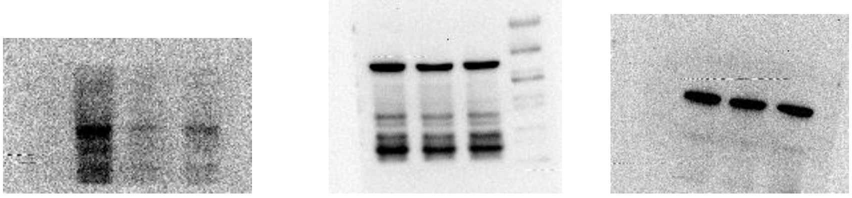

USP8 PD-L1 Tubulin

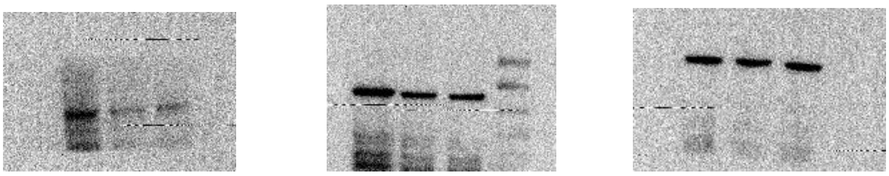

Figure 8A

IP_His-PD-L1_IB_HA-Ub

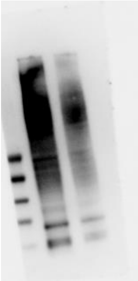

IP_ His-PD-L1_IB_ His-PD-L1

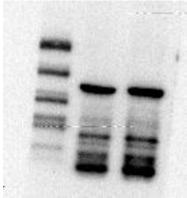

Input_ His-PD-L1

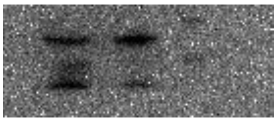

Input_USP8 or Input_Flag-USP8

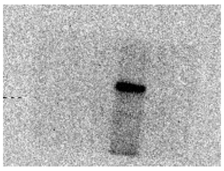

Input_ Tubulin

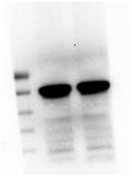

Figure 8B

IP_PD-L1_IB_Ub

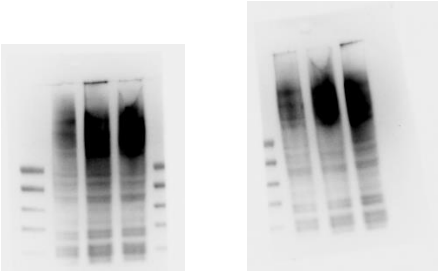

IP_ PD-L1_IB_ PD-L1

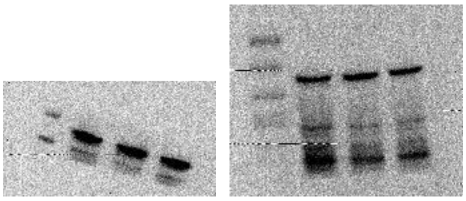

Input_ PD-L1

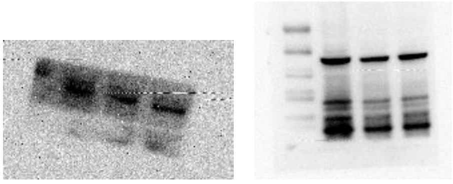

Input_USP8

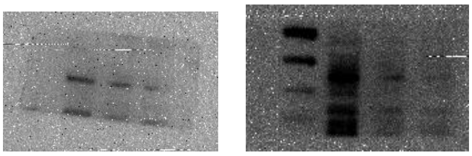

Input_ Tubulin

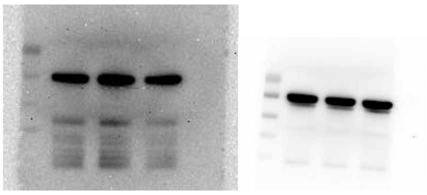

Figure 8C

HA-Ub Flag-USP8 His-PD-L1

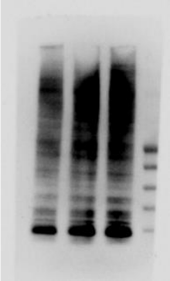

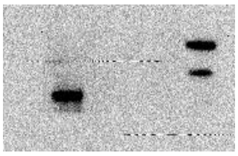

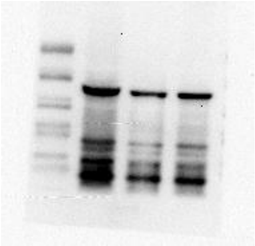

Figure 8D

IP_PD-L1_IB_Ub

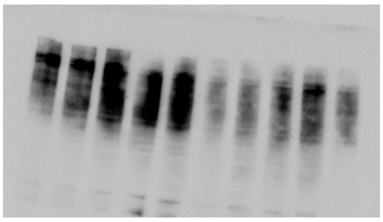

IP_ PD-L1_IB_ PD-L1

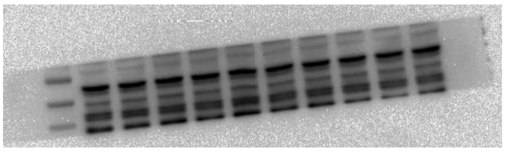

Input_ PD-L1

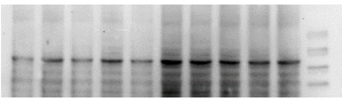

Input_USP8

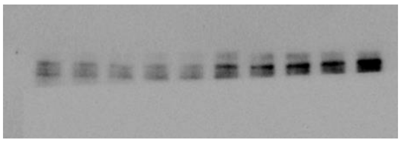

Input_Tubulin

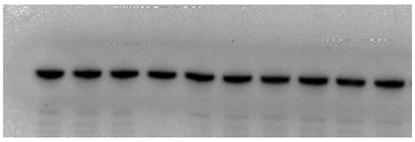

Figure 8E

IP_PD-L1_IB_Ub

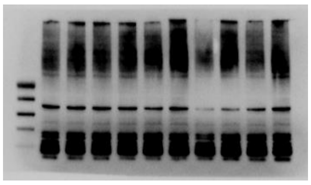

IP_ PD-L1_IB_ PD-L1

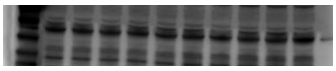

Input_ PD-L1

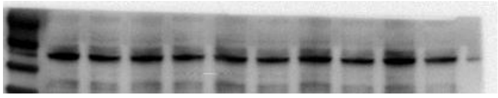

Input_USP8

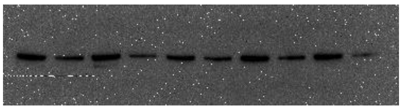

Input_Tubulin

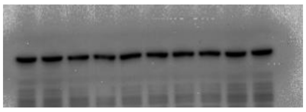

Figure 8F

IP_His-PD-L1_IB_HA-Ub

IP_ His-PD-L1_IB_ His-PD-L1

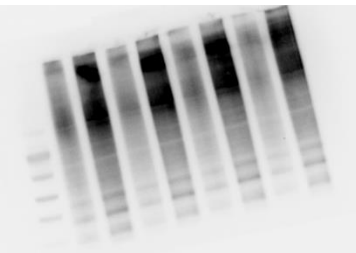

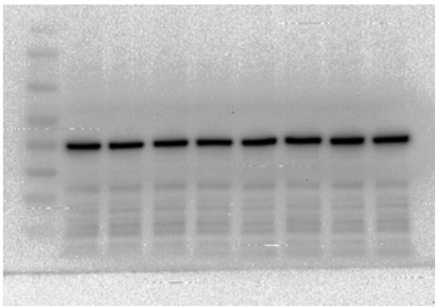

Input_ His-PD-L1

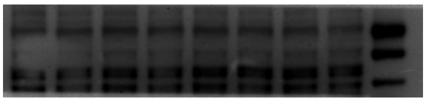

Input_Flag-USP8

Input_Tubulin

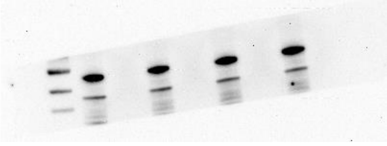
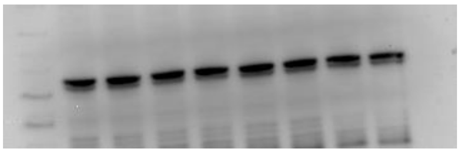

IP_His-PD-L1_IB_HA-Ub

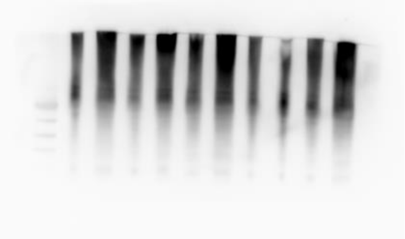

IP_ His-PD-L1_IB_ His-PD-L1

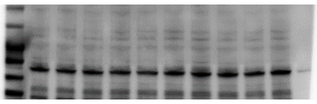

Input_ His-PD-L1

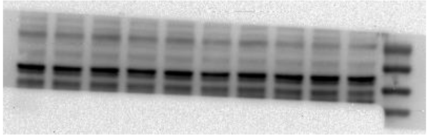

Input_Flag-USP8

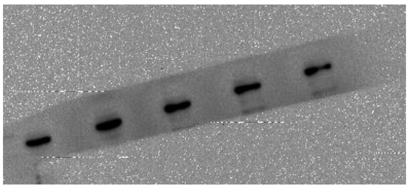

Input_Tubulin

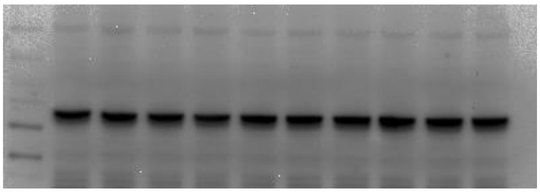

Figure 8G

USP8

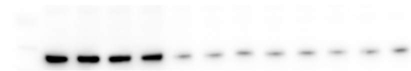

PD-L1

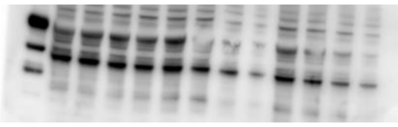

Tubulin

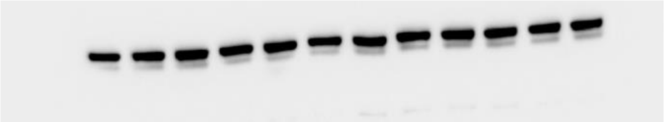

USP8

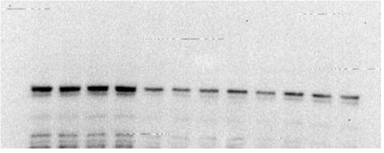

PD-L1

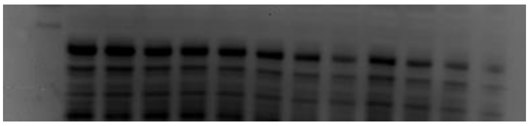

Tubulin

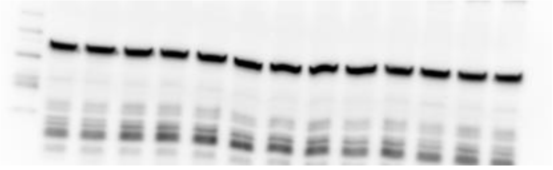

Figure 8H

PD-L1

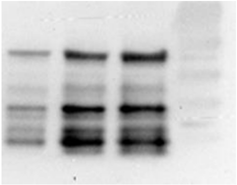

Tubulin

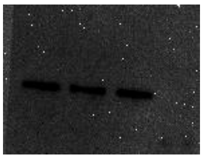

PD-L1

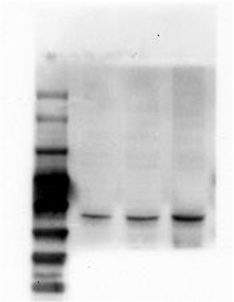

Tubulin

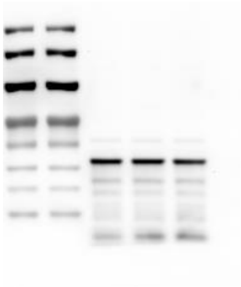

Figure S2F

PCNA

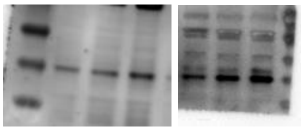

Cyclin D1

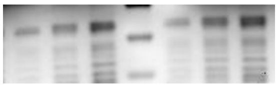

β-actin

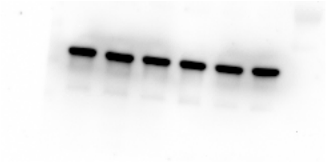

Figure S2H

N-Cadherin

E-Cadherin

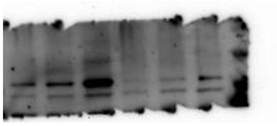

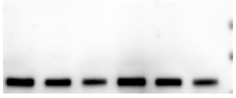

Vimentin β-actin

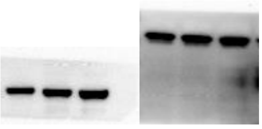

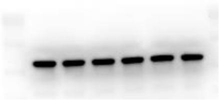

Figure S3E(DU145)

TEAD1 and β-actin

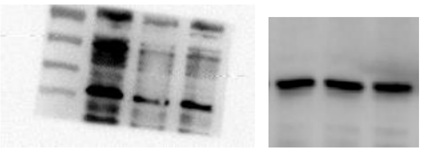

TEAD2 and β-actin

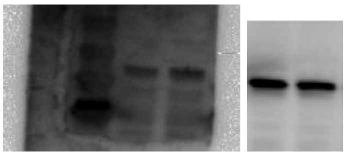

TEAD3 and β-actin

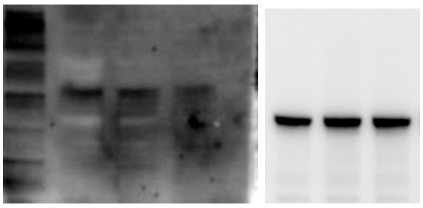

TEAD4 and β-actin

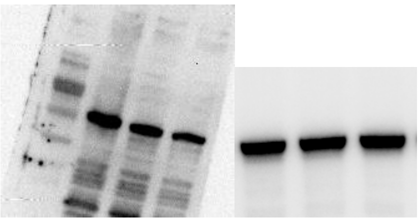

Figure S3E(PC-3)

TEAD1 and β-actin

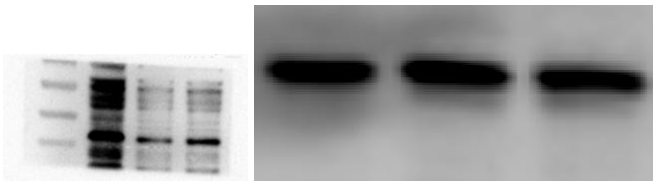

TEAD2 and β-actin

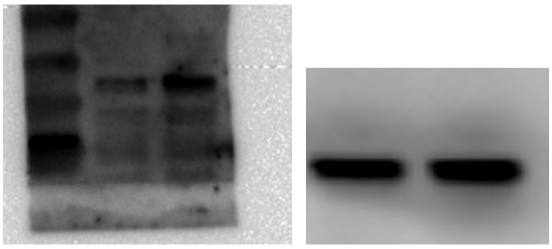

TEAD3 and β-actin

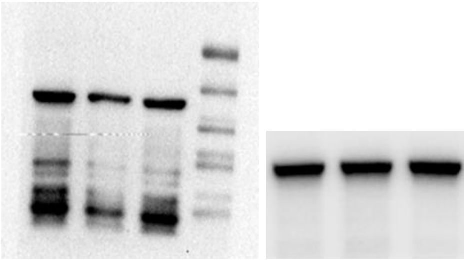

TEAD4 and β-actin

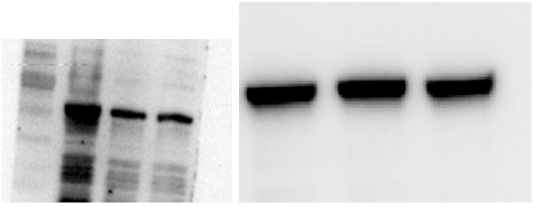

Figure S4B

USP8 β-actin

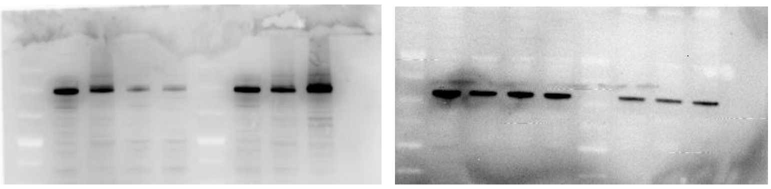

USP8 β-actin

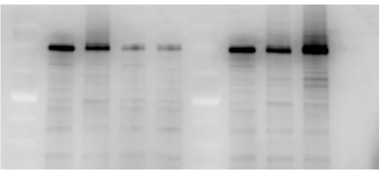

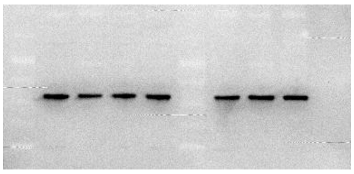

Figure S5A

IP_NBR1_IB_Ubi IP_NBR1_IB_NBR1

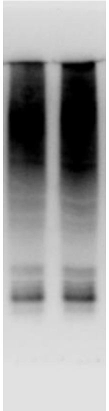

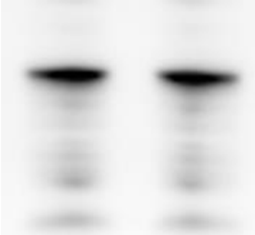

Input_NBR1 Input_TRIM21 Input_β-Actin

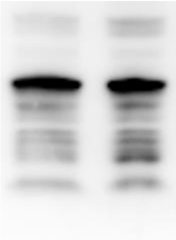

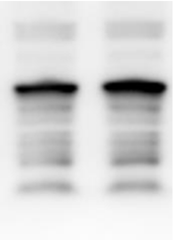

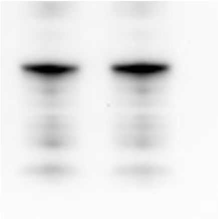

Figure S5B

Du145 NBR1 Du145 β-actin

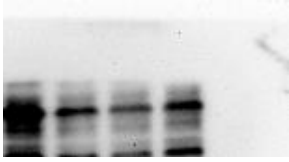

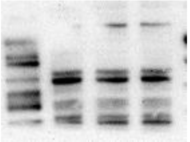

PC-3 NBR1 PC-3 β-actin

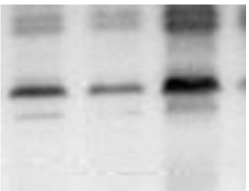

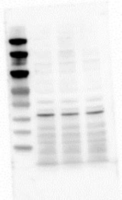

Figure S5C

NBR1

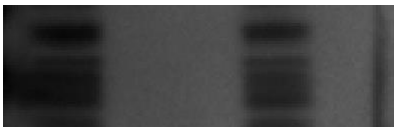

TRIM21

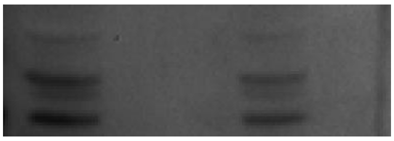

UBR5

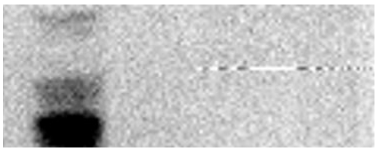

HERC2

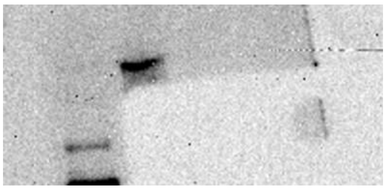

MIB1

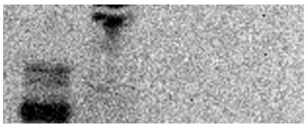

RBBP6

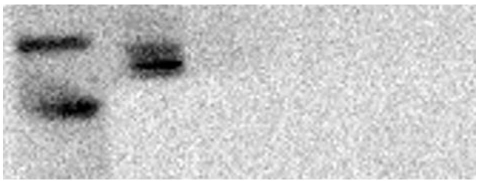

Figure S5E

IP_NBR1_IB_Ubi

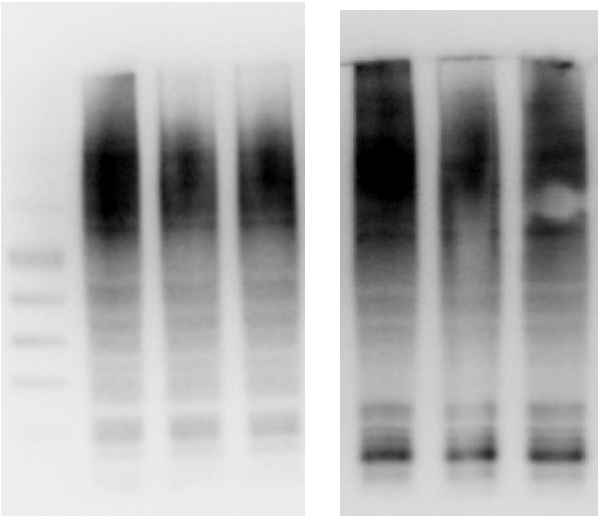

IP_NBR1_IB_NBR1

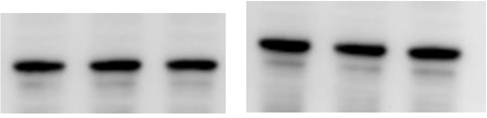

Input_NBR1

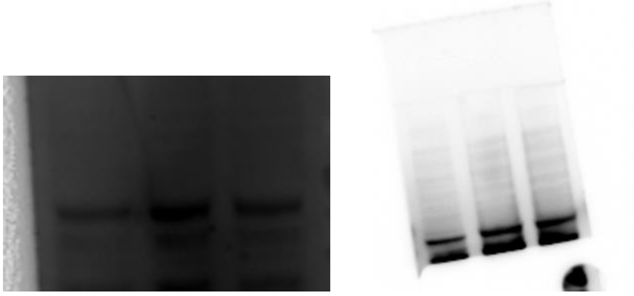

Input_TRIM21

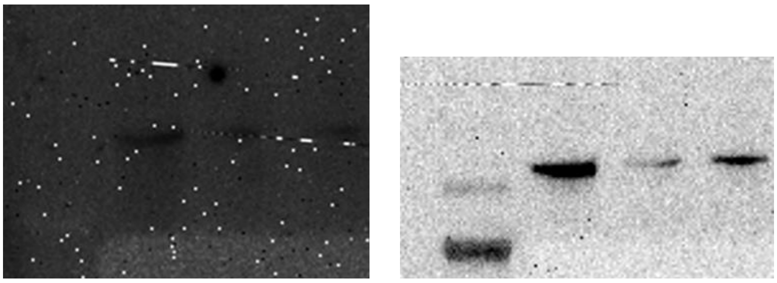

Input_β-Actin

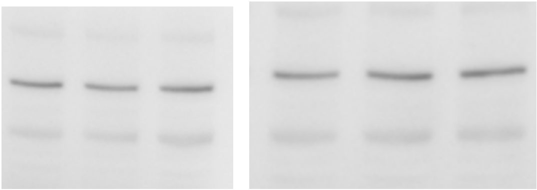

Figure S5F

NBR1

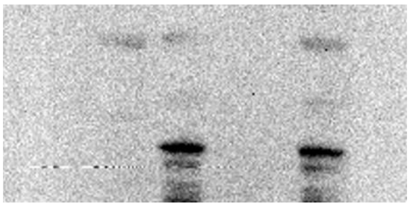

P62

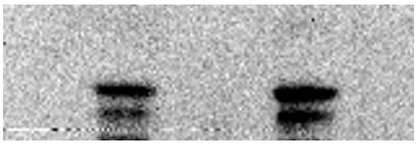

TAX1BP1

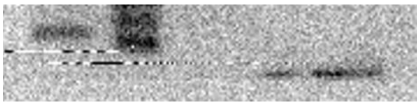

Figure S6A

IP_NBR1_IB_ Ub (RM-1) IP_ NBR1_IB_ NBR1(RM-1)

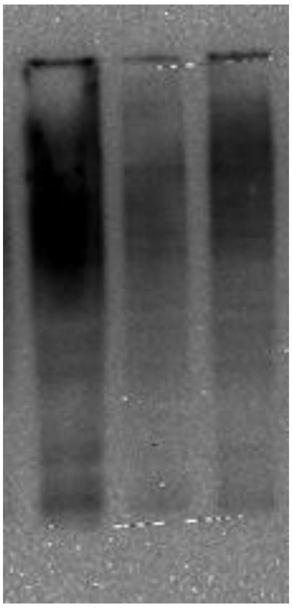

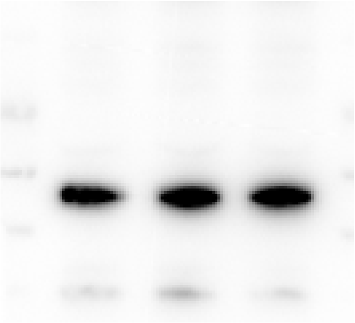

Input_ NBR1 (RM-1)

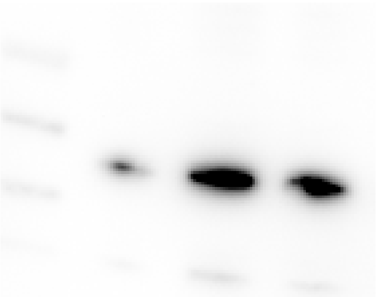

Input_USP8(RM-1) Input_ tubulin(RM-1)

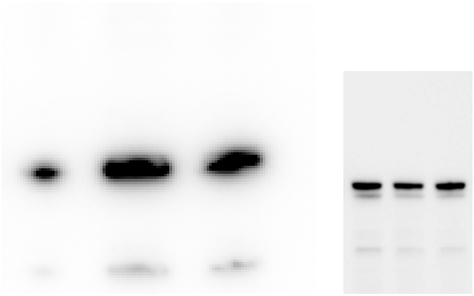

Figure S6B

IP_NBR1_IB_K63-Ub (DU145 and PC-3)

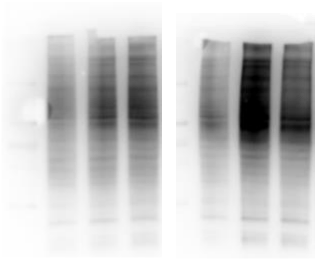

IP_ NBR1_IB_ NBR1(DU145 and PC-3)

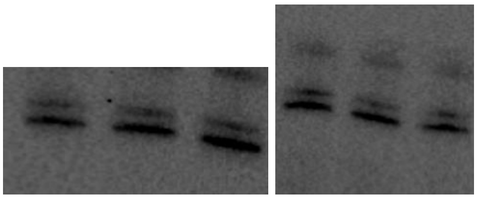

IP_NBR1_IB_K48-Ub (DU145 and PC-3)

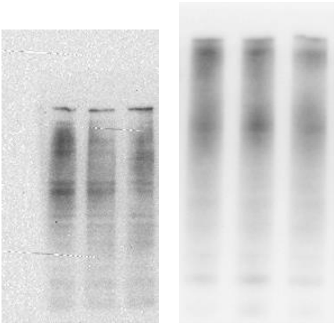

Input_ NBR1(DU145 and PC-3)

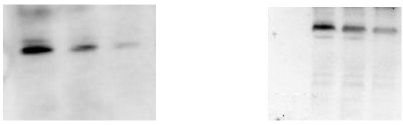

Input_USP8(DU145 and PC-3)

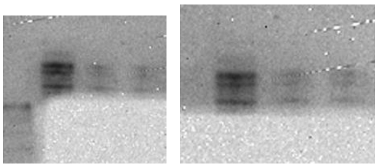

Input_ β-actin

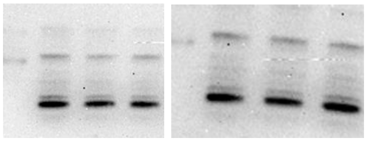

Figure S8

PC-3 tubulin

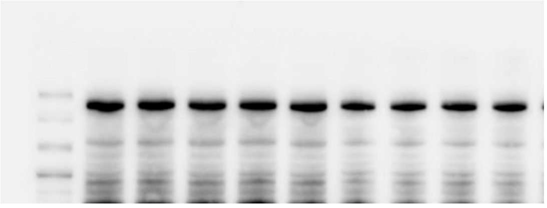

PC-3 MHC-1

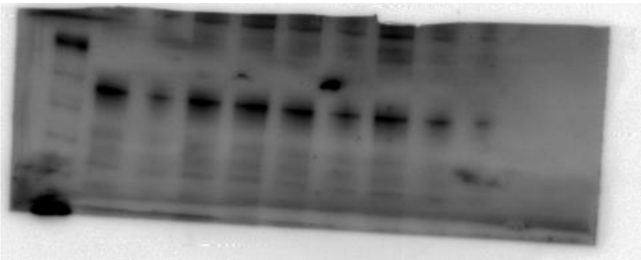

DU145 tubulin

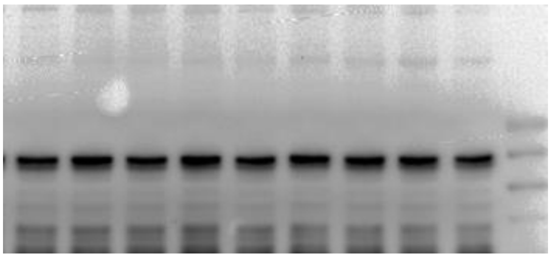

DU145 MHC-1

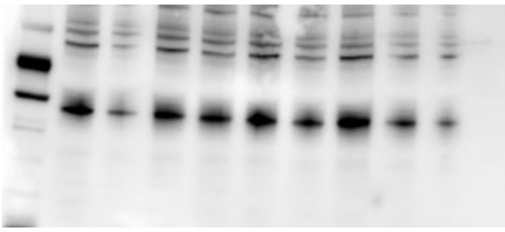

Figure S9A

IP_PD-L1_IB_ Ub (RM-1)

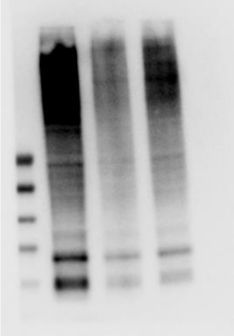

IP_ PD-L1_IB_ PD-L1(RM-1)

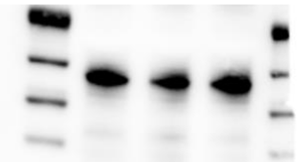

Input_ PD-L1 (RM-1)

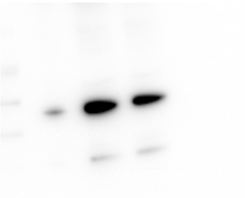

Input_USP8 (RM-1) Input_ Tubulin (RM-1)

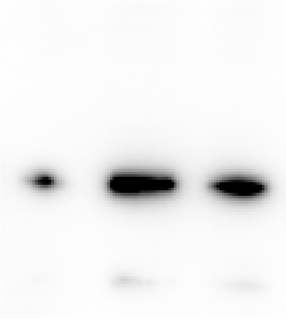

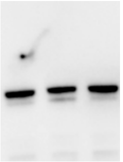

IP_PD-L1_IB_K48-Ub (DU145) IP_ PD-L11_IB_ PD-L1 (DU145)

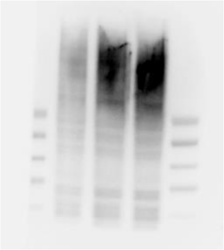

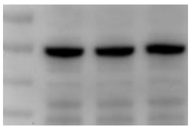

Input_ PD-L1 (DU145) IP_PD-L1_IB_K63-Ub (DU145)

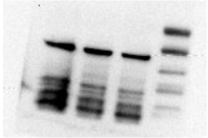

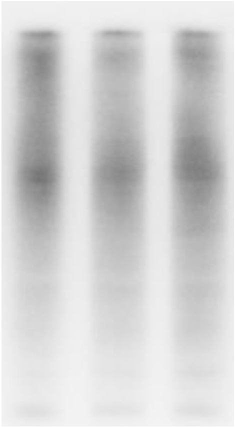

Input_USP8 (DU145) Input_ Tubulin(DU145)

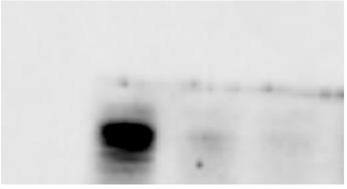

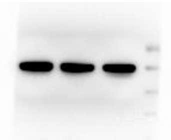

IP_PD-L1_IB_K48-Ub (PC-3)   IP_ PD-L11_IB_ PD-L1 (PC-3)

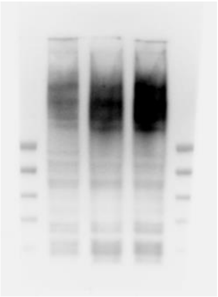

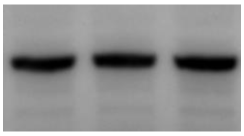

Input_ PD-L1(PC-3)   IP_PD-L1_IB_K63-Ub (PC-3)

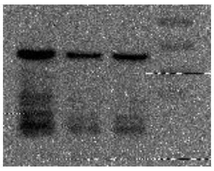

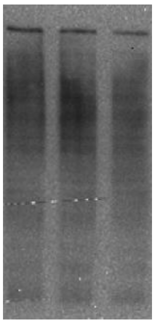

Input_USP8 (PC-3)   Input_ Tubulin(PC-3)

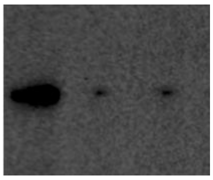

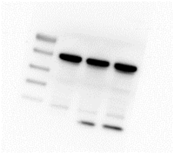

Supplement: Supplementary file 13 — Original data of WB [file 41419_2025_7736_MOESM13_ESM.pdf]
